# The Dimerization and Oligomerization of Alkenes Catalyzed with Transition Metal Complexes: Catalytic Systems and Reaction Mechanisms

**DOI:** 10.3390/molecules29020502

**Published:** 2024-01-19

**Authors:** Lyudmila V. Parfenova, Almira Kh. Bikmeeva, Pavel V. Kovyazin, Leonard M. Khalilov

**Affiliations:** Institute of Petrochemistry and Catalysis, Ufa Federal Research Center, Russian Academy of Sciences, 141 Prospekt Oktyabrya, Ufa 450075, Russia

**Keywords:** dimerization, oligomerization, transition metal catalysis, metal hydrides, reaction mechanisms

## Abstract

Dimers and oligomers of alkenes represent a category of compounds that are in great demand in diverse industrial sectors. Among the developing synthetic methods, the catalysis of alkene dimerization and oligomerization using transition metal salts and complexes is of undoubted interest for practical applications. This approach demonstrates substantial potential, offering not only elevated reaction rates but also precise control over the chemo-, regio-, and stereoselectivity of the reactions. In this review, we discuss the data on catalytic systems for alkene dimerization and oligomerization. Our focus lies in the analysis of how the activity and chemoselectivity of these catalytic systems are influenced by various factors, such as the nature of the transition metal, the ligand environment, the activator, and the substrate structure. Notably, this review particularly discusses reaction mechanisms, encompassing metal complex activation, structural and dynamic features, and the reactivity of hydride intermediates, which serve as potential catalytically active centers in alkene dimerization and oligomerization.

## 1. Introduction

The dimers and oligomers of alkenes belong to a broad class of compounds that are in high demand across various industrial sectors. Typically, they are used as comonomers in ethylene polymerization and serve as raw materials for the production of adhesives, surfactants, flavors, synthetic fuel additives, and more [1,2,3,4,5,6]. Alkene oligomers are synthesized catalytically using various methods, including heterogeneous acid catalysis with the use of phosphoric acid on silica, ion exchange resins, silica-aluminas, zeolites, etc., where the classical mechanism involving carbocations (carbenium pathway) is realized [6,7,8]. Another way to synthesize oligomers involves transition metal catalysis (Zr, Ti, Hf, Ni, Co, Fe, V, and Ta) in which the Cossee–Arlman mechanism is implemented [9]. Metal-catalyzed processes, such as the oligo- and polymerization of ethylene on chromium catalysts (Philips), the preparation of linear α-olefins via ethylene oligomerization on a nickel catalyst (SHOP = shell higher olefin process), the oligomerization of light alkenes into C4–C10 olefins using AlphaButol, AlphaHexol, Dimersol, and Difasol process technologies, and others, were developed to produce olefin oligomers [10,11,12,13,14]. The oligomerization of alkenes (1-butene and 1-hexene) synthesized from renewable plant raw materials to obtain jet and diesel fuels is also attracting the attention of the scientific community [6,15].

Among the developing methods with significant potential for advancement and practical implementation is the catalysis of alkene dimerization and oligomerization using Ti subgroup metal salts and complexes, which provide high reaction rates and effective control of their chemo-, regio-, and stereoselectivity. The classical heterogeneous Ziegler–Natta catalysis is widely used in the production of polyethylene and polypropylene [16,17,18,19,20,21]. The discovery of metallocenes [22], along with organoaluminum [23,24,25] and boron activators [22,26,27], enabled the process to be transferred from a heterogeneous medium to a homogeneous one, which provided undoubted positive effects—such as an increase in the activity and the possibility to effectively control stereoselectivity—and allowed for a detailed study to be conducted on the reaction mechanisms. Homogeneous systems effectively catalyze alkene di-, oligo-, and polymerization [1,28,29,30], as well as the hydro-, carbo-, and cyclometalation of olefins and acetylenes [31,32,33,34,35], which can not only be considered as the methods of multiple bond functionalization but also as the initial stages of chain growth in the oligo- and polymerization processes. 

**Scheme 1:**
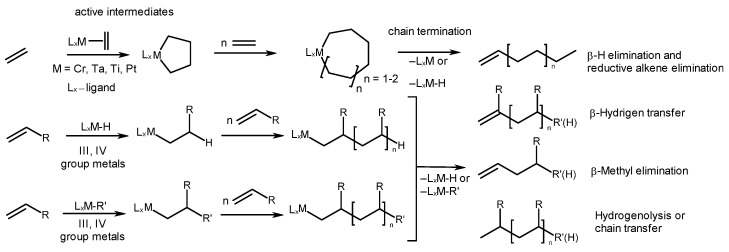
Alkene oligomerization catalyzed with transition metal complexes: active centers and types of products.

Information concerning the catalytic alkene oligomerization and the types of products it produces can be seen in Scheme 1. The nature of the active reaction centers determines the structural types of the resulting oligomers and the regio- and stereoselectivity of a substrate’s insertion. In the processes presented in Scheme 1, hydride, alkyl, or alkene intermediates act as active reaction centers, which, in the early stages, facilitate alkene hydro-, carbo-, or cyclometalation, respectively. Chain termination occurs through the elimination of the oligomeric product, generating metal hydrides, or through the transfer of the growing chain to an organometallic cocatalyst or alkene. Metal hydrides, therefore, can serve as dominant reaction centers in these catalytic systems. 

Recent reviews on catalytic ethylene, propylene, and higher olefin oligomerization discussed the chemical methodologies, possible reaction mechanisms, techniques used to study the structure and physicochemical properties of oligomers, the practical implementation of these processes in the industry, and the potential applications for the resulting products [1,3,36,37,38,39,40,41,42].

This review paper considers catalytic systems based on metal complexes for the synthesis of alkene dimers and oligomers in the context of the dependence of the activity and chemoselectivity of catalytic systems on the nature of the transition metal, ligand environment, activator, and substrate structure. This review pays particular attention to the reaction mechanisms, including the metal complex activation, the structural and dynamic features, and the reactivity of hydride intermediates, as potential catalytically active centers in alkene dimerization and oligomerization.

## 2. Catalytic Synthesis of Terminal Alkene Dimers and Oligomers

**Scheme 2:**
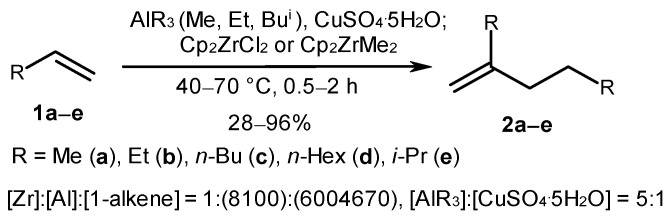
Alkene dimerization under the action of a catalytic system: Cp_2_ZrCl_2_ (Cp_2_ZrMe_2_)-AlR_3_ (R = Me, Et, and Bu^i^)-CuSO_4_·5H_2_O [43].

In the literature, significant attention is given to Ti subgroup metal complexes, the use of which, in homogeneous catalytic systems, ensures alkene dimerization, oligomerization, and polymerization with high yields and high chemo- and stereoselectivity [1,5,40]. The selective transformation of α-olefins (propene (**1a**), 1-butene (**1b**), 1-hexene (**1c**), 1-octene (**1d**), and 3-methyl-1-butene (**1e**)) into vinylidene dimers (**2a**–**e**) under the action of a catalytic system consisting of Cp_2_ZrCl_2_ (**3**) or Cp_2_ZrMe_2_ (**4**) and aluminoxane, obtained in situ through the reaction of AlR_3_ (R = Me, Et, and Bu^i^) with CuSO_4_·5H_2_O, was reported in one of the first works on this topic (Scheme 2) [43]. Dimeric products were obtained with a selectivity of up to 96% during the reaction performed at 40–70 °C for 0.5–2 h with the following reagent ratio: [Zr]:[Al]:[1-alkene] = 1:(8–100):(600–4670). The highest alkene conversion rate and selectivity towards the dimerization were achieved in the reaction, which was catalyzed with Cp_2_ZrCl_2_ and methylaluminoxane (MAO).

**Scheme 3:**
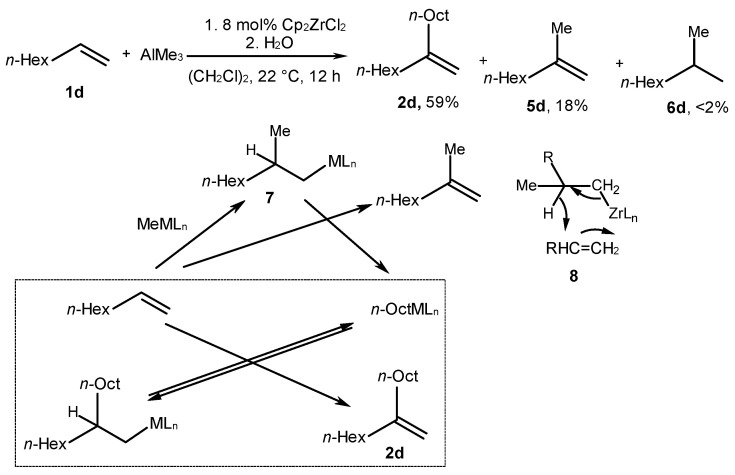
Reaction of 1-octene with AlMe_3_ in the presence of Cp_2_ZrCl_2_ in 1,2-dichloroethane and probable mechanism [44].

A dimeric product (**2d**) was obtained with a yield of 59% in the reaction of 1-octene with AlMe_3_ in the presence of Cp_2_ZrCl_2_ (**3**) in 1,2-dichloroethane at 22 °C for 12 h (Scheme 3) [44]. It was assumed that the initial stage of the reaction was the alkene carbometalation and the formation of metal alkyl **7**, which hydrometalates 1-octene through state **8**. As a result, 2-methyl-1-octene and the hydrometalation product, n-OctML_n_, are accumulated in the mixture. The latter reacts with 1-octene via carbometalation to provide 2-(n-hexyl)-1-decyl metal that hydrometalates 1-octene to form n-OctML_n_ and dimer **2d** (Scheme 3).

**Scheme 4:**
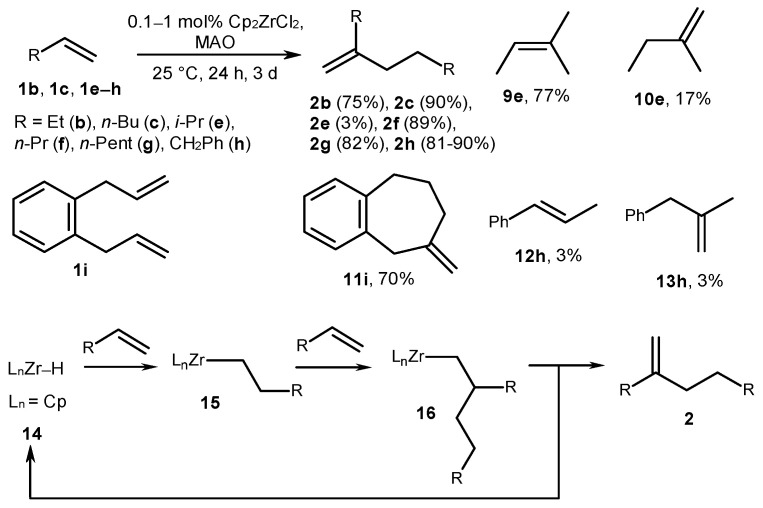
Alkene dimerization in the presence of catalytic system (Cp_2_ZrCl_2_-MAO) and probable reaction mechanism [45,46].

Furthermore, terminal alkenes **1b**, **1c**, and **1e**–**h** were dimerized in the presence of the catalytic system Cp_2_ZrCl_2_-MAO at an Al/Zr ratio of 1:1 and a room temperature of 25 °C for 24 h with the product yield of 80–90% (Scheme 4) [45,46]. 3-Methyl-1-butene (**1e**) provided a mixture of 2-methyl-2-butene (**9e**, 77%), 2-methyl-1-butene (**10e**, 17%), and 5-methyl-2-(methylethyl)-1-hexene (**2e**, 3%). The reaction of *o*-diallylbenzene (**1i**) with Cp_2_ZrCl_2_ and MAO at an Al/Zr ratio of 4:1 for 3 days provided a cyclic product, methylenecycloheptane **11i**, with a 70% yield. 

The mechanism proposed in [45,46] for the dimerization reaction implies the insertion of 1-alkene into a Zr-H bond of zirconocene hydride **14** to obtain Zr-alkyl complex **15**, which then carbometalates the second alkene molecule, producing alkyl derivative **16**. Subsequent β-H elimination in alkyl complex **16** provides dimer **2** and hydride complex **14** (Scheme 4). It is noted that the presence of chlorine in a catalytic system is an important factor for the dimerization reaction. In confirmation, a higher oligomer formation was seen in the presence of Cp_2_ZrMe_2_ (**4**) as a catalyst and MAO (without Cl atoms) was given. The chlorine atom probably ensures the fast β-H elimination, but not the growth of an alkyl chain [46].

**Scheme 5:**
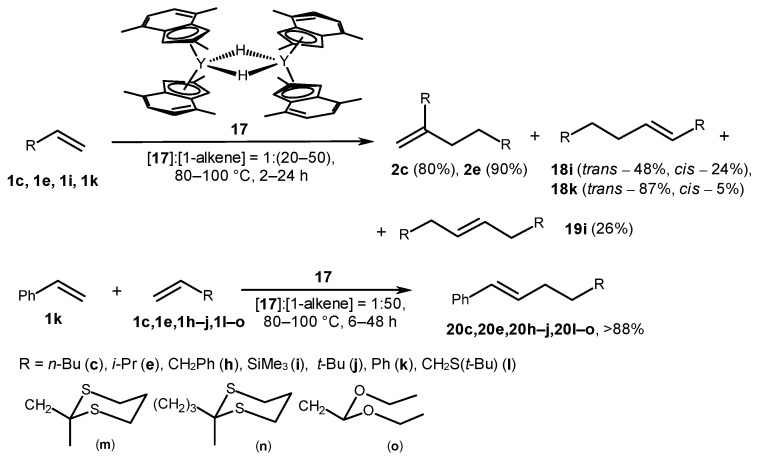
α-Olefin homo- and codimerization, catalyzed with the following hydride complex: [(2,4,7-Me_3_-Ind)_2_Y(μ-H)]_2_ [47].

A dimeric hydride complex, [(2,4,7-Me_3_-Ind)_2_Y(μ-H)]_2_ (**17**) (Scheme 5), appeared to be an effective catalyst for the homodimerization of various α-olefins [47]. The reaction was performed in benzene at 80–100 °C for 2–24 h, and there was a 20–50-fold molar excess of α-olefins. The head-to-tail dimerization was observed in the case of 1-hexene (**1c**) and 3-methyl-1-butene (**1e**) with a selectivity of >98%.

**Scheme 6:**
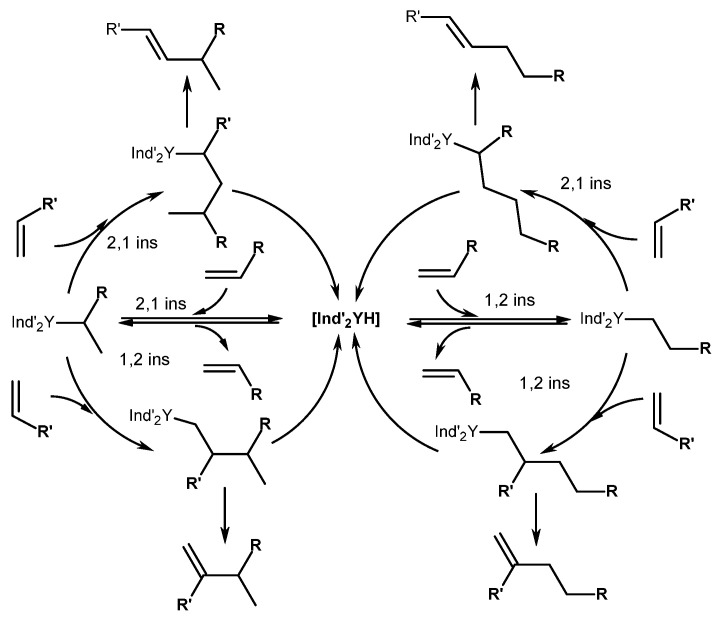
Proposed mechanism of α-olefin homo- and codimerization, catalyzed with [(2,4,7-Me_3_-Ind)_2_Y(μ-H)]_2_ (**17**) [47].

The reaction runs through sequential 1,2-insertion followed by β-H elimination (Scheme 6) [47]. The homodimerization of trimethylvinylsilane (**1i**) and styrene (**1k**) occurred, forming head-to-head products. The reactions presumably proceed through an initial 1,2-insertion into the Y-H bond, followed by a subsequent 2,1-coordination and β-H abstraction. Olefins **1l**–**o**, containing heteroatoms, 3,3-dimethyl-1-butene (**1j**), and allylbenzene (**1h**), did not undergo homodimerization under the reaction conditions. In the reaction with **1h**, C-H activation arose, resulting in the formation of a catalytically inactive allyl complex, Ind’_2_Y(η^3^-CH_2_CHCHPh). The reaction of **17** with **1l**–**o** provided stable alkyl complexes that deactivated the catalyst.

Complex **17** also showed activity in the styrene codimerization with alkenes (H_2_C=CHR) that produced trans-1-phenyl-4-alkylbut-1-enes (**20**) with more than an 88% yield at 80–100 °C (Scheme 5). The reaction probably occurred through the 1,2-coordination of α-olefin into the Y-H bond, followed by a subsequent 2,1-insertion of styrene into the Y-C bond of the alkyl intermediate and β-H elimination (Scheme 6). Heteroatom-containing olefins **1l**–**o** readily formed head-to-head codimers with styrene. However, these substrates exhibited lower reactivity, and the accompanying formation of the styrene homodimer was observed.

**Scheme 7:**
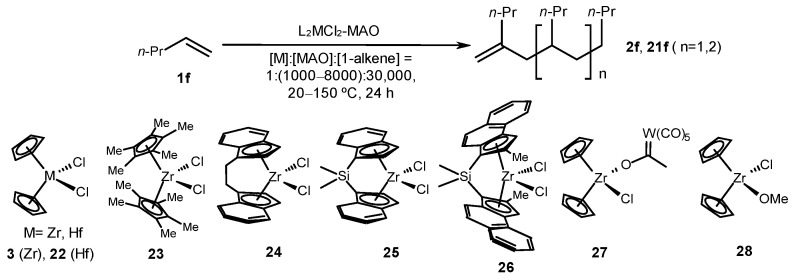
1-Pentene oligomerization, catalyzed with complexes **2** and **22**–**28 [48,49]**.

Complexes of various structures were subsequently tested in the reactions to find selective catalysts for alkene dimerization and oligomerization. For example, 1-pentene (**1f**) was transformed into oligomers in the presence of catalytic systems based on *bis*-cyclopentadienyl complexes **3**, **22**, and **23** and MAO in a ratio ([Zr]:[MAO]:[substrate] = 1:1000:30,000) at 60 °C for 24 h in toluene (Scheme 7) [48]. Oligomeric products with low molecular weights were obtained: dimers (25%), trimers (18%), and tetramers (14%). The use of catalysts with *ansa*-indenyl ligands (EBI)ZrCl_2_ (**24**) and (SBI)ZrCl_2_ (**25**) led to the formation of isotactic poly(1-pentene) (M_N_ = 1700–4400 g mol^−1^, PDI = 4.75–6.41). Further studies on the catalytic properties of complexes **3** and **22**–**28** at a reagent ratio ([metallocene]:[MAO]:[1-alkene] = 1:(1000–8000):30,000) at 20–150 °C demonstrated the dependence of the reaction chemoselectivity upon the metallocene structure [49]. The reaction of 1-pentene, catalyzed with complexes **22** and **23** and MAO ([Zr]:[MAO] = 1:1000), resulted in an atactic polypentene, whereas *ansa*-indenyl complexes **24**–**26** provided an isotactic polymer with stereoselectivities of 0.91, 0.45, and 0.64 (mmmm), respectively. The polymer with the highest molecular weight (M_W_ = 149,000, PDI = 1.85–2.08) was obtained by using catalyst **26**. The reaction, which was catalyzed with cyclopentadienyl complexes **3**, **27**, and **28**, under these conditions, afforded the oligomeric products with 2–4 units. In this case, the highest conversion (50%) was achieved in the presence of bimetallic complex **27**, whereas the yields of dimers, trimers, and tetramers were 10, 20, and 20%, respectively. An increase in the amount of MAO to 6000 eq. in the case of complex **3** caused an increase to 80% in the alkene conversion, and the yields of dimers, trimers, and tetramers increased to 15, 30, and 35%, respectively (Scheme 7) [49].

**Scheme 8:**
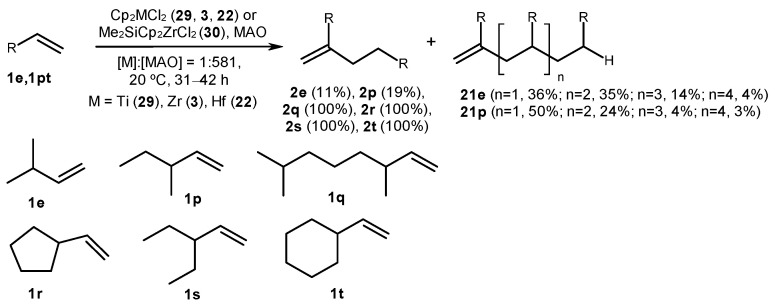
Oligomerization of branched *α*-olefins, catalyzed with Cp_2_MCl_2_ (M = Ti (**29**), Zr (**3**), and Hf (**22**)) or Me_2_SiCp_2_ZrCl_2_ (**30**); yields are given for catalyst **30** [50].

Branched *α*-olefins were regioselectively dimerized at 20 °C in toluene for 3–142 h upon the action of Cp_2_MCl_2_ (M = Ti (**29**), Zr (**3**), and Hf (**22**)) or Me_2_SiCp_2_ZrCl_2_ (**30**) and MAO at the following ratio: [M]:[MAO] = 1:581 (Scheme 8) [50]. Complex **30** with Me_2_Si-bonded cyclopentadienyl ligands showed the highest activity and regioselectivity, providing dimers **2q**–**t** with yields of up to 100%. 3-Methyl-1-butene (**1e**) and 3-methyl-1-pentene (**1p**) provided dimers with yields of 11 and 19%, as well as oligomeric products **21e** and **21p**, correspondingly.

**Scheme 9:**
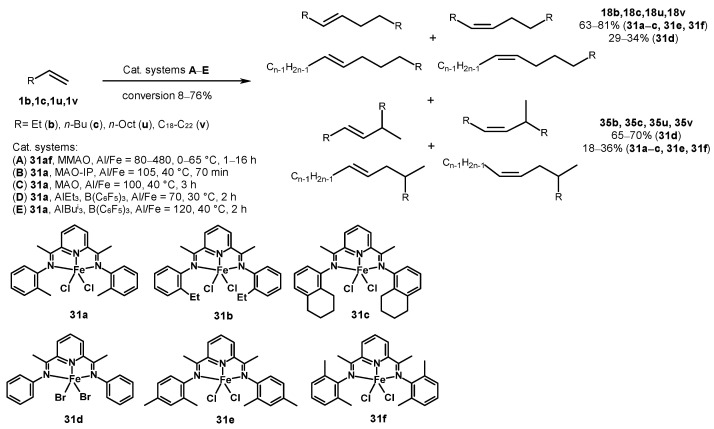
Dependence of the type of alkene dimerization products on the post-metallocene structure [51].

As a rule, the application of other transition metal complexes changes the regioselectivity of a reaction. For example, pyridine bis(imine) iron complexes **31a**,**b**, upon activation with MAO, MMAO, or AlR_3_ (R = Et, Bu^i^)-B(C_6_F_5_)_3_ (Al/Fe = 70–480), demonstrate the ability to dimerize various α-olefins, such as **1b**, **1c**, **1u**, and **1x** (Scheme 9) [51]. This results in the formation of a mixture of linear olefin dimers **18b**, **18c**, **18u**, and **18x** with internal double bonds (63–80%) and monomethyl-branched dimers **35b**, **35c**, **35u**, and **35x** (18–36%). Additionally, trisubstituted vinylidene (2-alkylalkenes) or α-olefinic products were detected in trace amounts. High alkene conversion up to 76% was achieved in the presence of **31a**–**c** and **31e** at 30–50 °C. The sterically less hindered complex **31d** provided monomethyl-branched dimers **35b**, **35c**, **35u**, and **35x**.

**Scheme 10:**
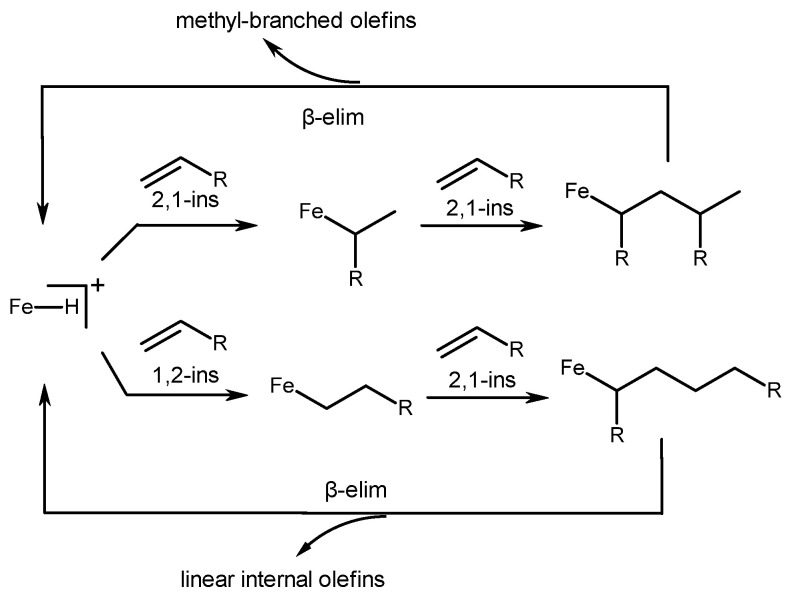
Mechanism of alkene dimerization, catalyzed with iron complexes **31a**–**d** [51].

The reaction mechanism consists of consecutive stages of the 1,2-insertion of the initial olefin into the Fe-H bond, followed by a 2,1-insertion of the second olefin (Scheme 10). Subsequent β-H elimination leads to the formation of linear dimers. Successive 2,1-insertions of alkenes and β-H eliminations produce Me-substituted dimers.

**Scheme 11:**
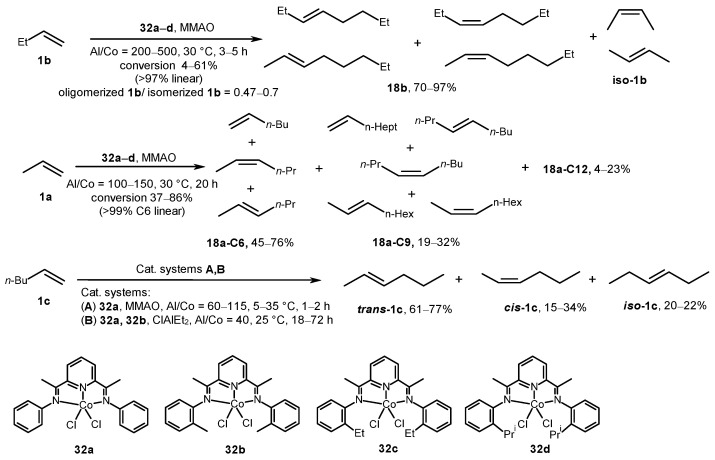
Pyridine bis(imine) cobalt complexes **32a**–**d** as catalysts of α-olefin dimerization [52].

Pyridine bis(imine) cobalt catalysts **32a**–**d**, when activated with MMAO (Al/Co = 200–500), dimerized α-olefins with lower productivity compared to similar iron systems (TON for 1-butene: 42,000 vs. 147,000 for Co and Fe, respectively) (Scheme 11) [52]. The main products were linear dimers (>97%) and butene isomers in an **18b**/**iso-1b** ratio of 0.47–0.7. In the dimerization of propylene, linear hexenes, nonenes, and dodecenes were obtained with turnovers exceeding 200,000 moles of propylene/mole Co (17,000 g oligomer/g Co complex). Complexes **32a** and **32b**, in combination with MMAO or EtAlCl_2_, induced the isomerization of 1-hexene. 

**Scheme 12:**
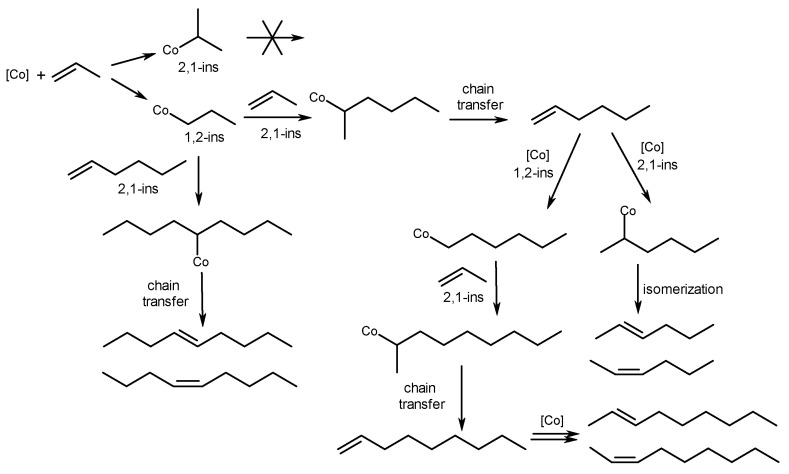
Mechanism of alkene dimerization, catalyzed with Co complexes **32a**–**d**.

The main stages of the stepwise reaction mechanism include a consecutive 1,2-insertion of olefin and a 2,1-insertion into Co-Alkyl, followed by chain termination to provide alkenes with internal and terminal double bonds (Scheme 12) [52].

**Scheme 13:**
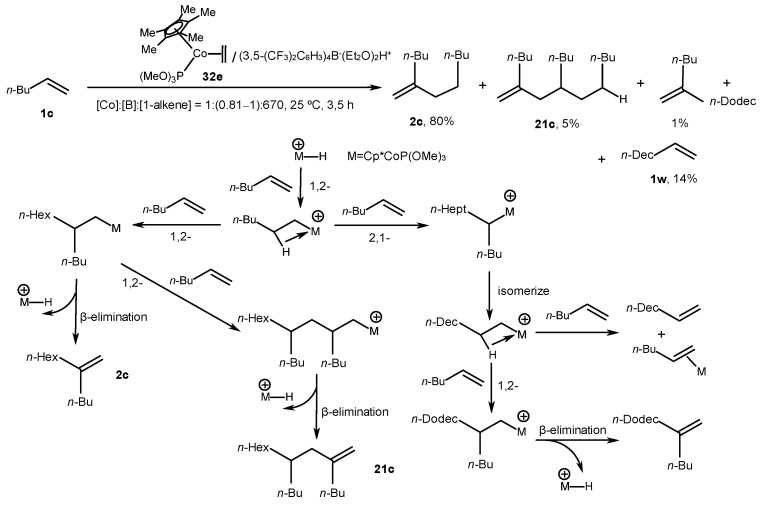
Transformations of terminal alkenes into dimers, which were catalyzed with complex **32e [53]**.

Nevertheless, mixed ethylene Co complex **32e** catalyzed the transformation of terminal alkenes into vinylidene dimers of a head-to-tail type with yields of 66–80% in the presence of an organoboron activator, HBArF, at a [Co]:[B]:[1-alkene] ratio of 1:0.81:670 in contrast to the post-metallocene catalysts **32a**–**d** (Scheme 13) [53]. Moreover, linear terminal alkene **1w** formed in the reaction with a yield of up to 14%, presumably due to the isomerization processes in intermediate alkyl Co complexes.

**Scheme 14:**
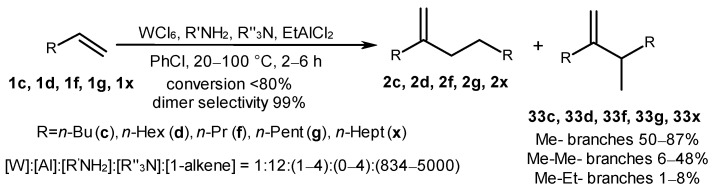
Alkene dimerization under the action of a catalytic system, WCl_6_/R′NH_2_/R″_3_N/EtAlCl_2_ [54].

α-Olefins **1c, 1d**, **1f**, **1g**, and **1x** undergo tail-to-tail dimerization under the action of a catalytic system, WCl_6_/R′NH_2_/R″_3_N/EtAlCl_2_, obtained in situ at a [W]:[R′NH_2_]:[R″_3_N]:[EtAlCl_2_]:[1-alkene] molar ratio of 1:(1–4):(0–4):12:(834–5000) to provide predominantly methyl-branched products (**33c**, **33d**, **33f**, **33g**, and **33x**) (Scheme 14) [54]. Alkene conversion at a level of 80% and high selectivity towards the dimerization were achieved (>99%) due to the optimal choice of a chlorine-containing organoaluminum activator (EtAlCl_2_) and a solvent, PhCl. This effect on the reaction initiation was attributed to the generation of bimetallic catalytically active centers with a W-Cl-Al bridge.

**Scheme 15:**
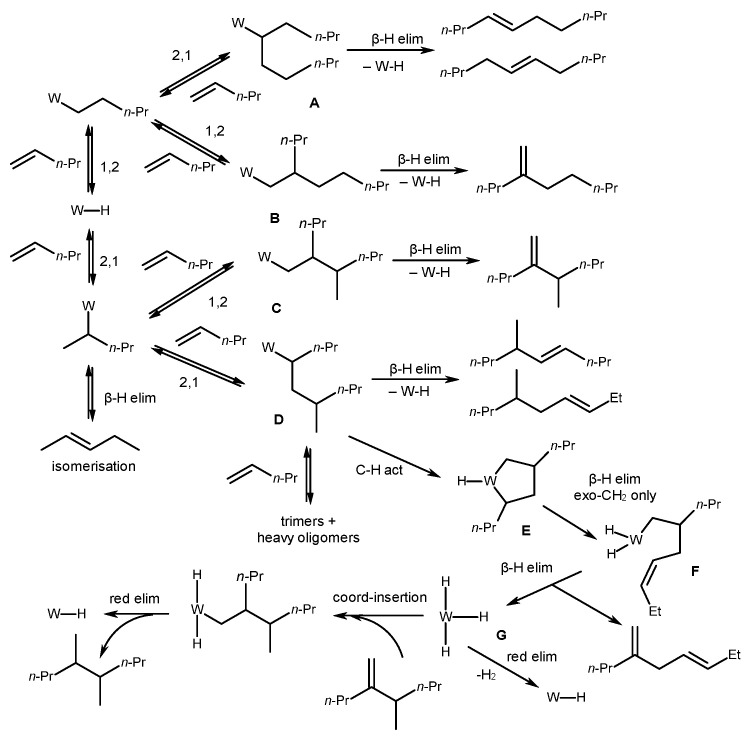
Mechanism of alkene dimerization under the action of a catalytic system, WCl_6_/R′NH_2_/R″_3_N/Et_2_AlCl [54].

Upon a detailed analysis of the reaction products using the example of 1-pentene dimers, it was demonstrated that fractions of linear C10 products (constituting only 0.1% of the dimer fraction) contain *trans*-5-decene, *cis*-4-decene, dienes, and decane (Scheme 15) [54]. The authors proposed a Cossee-type mechanism [9], noting that the initial insertion of an alkene occurs equally as 1,2- and 2,1-, followed by a subsequent regioselective alkene 1,2-coordination. Therefore, the dominant structures appear to be **B** and **C**, which provide the main reaction products.

**Scheme 16:**
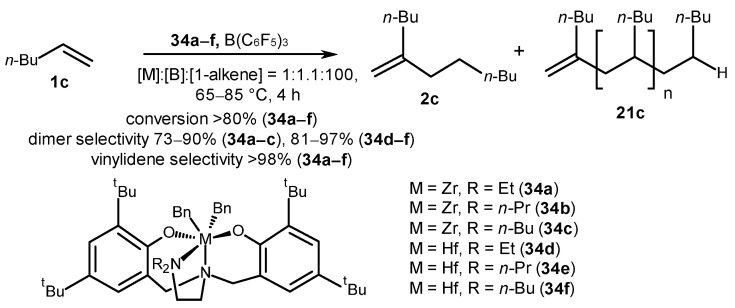
Post-metallocene Zr and Hf amino-bis(phenolate) complexes of [ONNO]-type as catalysts of 1-hexene oligomerization [55].

Low-molecular-weight oligomeric products, including 1-hexene dimers, were synthesized with high yields (73–97%) and selectivity (≥98%) in the presence of Zr and Hf post-metallocene complexes with amino-bis(phenolate) [ONNO] ligands and a neutral activator, B(C_6_F_5_)_3_, at 65–85 °C for 4 h and the following reagent ratio: [metallocene]:[B]:[1-hexene] = 1:1.1:100 (Scheme 16) [55]. The highest activity in the oligomerization was achieved in the presence of Zr catalysts **34a**–**c**; in this case, the molecular weights of the products corresponded to a typical Flory–Schulz distribution [56]. Hafnium catalysts **34d**–**f** showed lower activities in contrast to the zirconium analogs; however, they showed greater selectivity in dimerization. In addition, the molecular weight distribution of the products obtained in the presence of hafnium catalysts did not follow the Schultz–Flory distribution. The high selectivity in the formation of vinylidene dimers was explained by the prevalence of 1,2-alkene insertions into catalytically active centers, both in primary M-H and secondary M-Alkyl species. It was also noted that the chain termination rate for these systems exceeds the rate of chain propagation. In the case of regioerror, i.e., alkene 2,1-insertions, conversely, the chain propagation prevails because the elimination is practically impossible; therefore, chain termination via β-H elimination will occur when the 1,2-incorporation of an alkene takes place. The authors explain that deviations from the Schultz–Flory distribution are caused by the presence of two or more conformations of hafnium active centers, which have different activities towards alkene (the assumption was made from the ^1^H NMR spectra of the initial complexes depending on temperature). For zirconium analogs, it seems that either one isomer is characteristic, or the exchange between conformations is very fast (the energy barrier is small), so it does not significantly affect the distribution of oligomers.

**Scheme 17:**
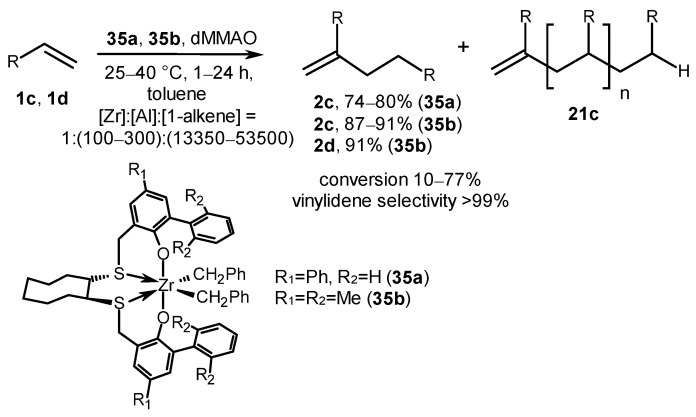
Post-metallocene Zr complexes of [OSSO] type as catalysts of alkene oligomerization [57].

A highly regioselective method for the oligomerization of 1-hexene and 1-octene was developed at relatively low catalyst loadings (0.0019–0.0075 mol%) using Zr complexes **35a** and **35b** with [OSSO]-type aryl-substituted bis(phenolate) ligands and modified methylaluminoxane (dMMAO) (Scheme 17) [57]. The catalytic system predominantly produced dimers with terminal vinylidene groups (74–91%) and trimers (8–11%) at 25–40 °C for 1 h with the following reagent ratio: [Zr]:[Al]:[1-alkene] = 1:(100–300):(13,350–53,500). The TOF values were adjusted by changing the structure of an aryl substituent R_1_ at the *ortho* position of a phenolate moiety of the [OSSO] ligand and the number of dMMAO equivalents used. The highest TOF value (up to 11,100 h^−1^) was observed for phenyl-substituted precatalyst **35a**. The authors explained the low alkene conversion (10–77%) in the presence of **35a** and **35b** with the deactivation of Zr-H active species during oligomerization.

**Scheme 18:**
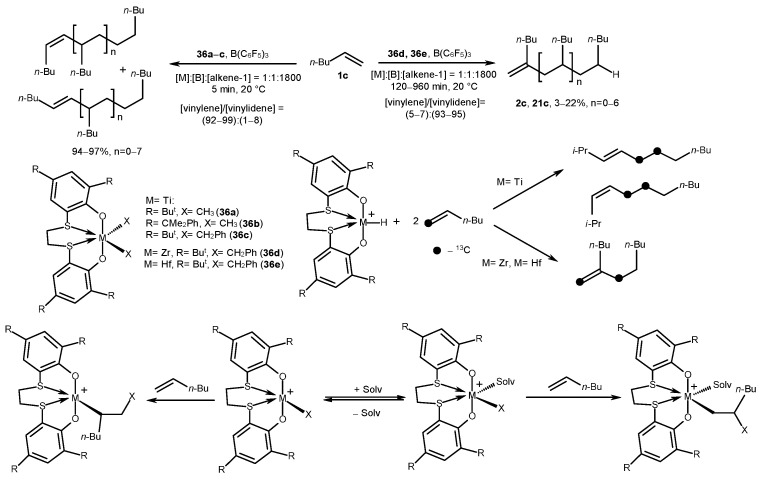
Post-metallocene bis-phenolate Zr complexes of [OSSO] type as catalysts of alkene oligomerization [58].

Bis-phenolate titanium complexes **36a**–**c**, activated by B(C_6_F_5_)_3_ ([Ti]/[B] = 1), catalyzed the transformation of 1-hexene into vinylene oligomers with high yield (up to 97%) and selectivity (99%) (Scheme 18) [58]. Zirconium (**36d**) and hafnium analogs (**36e**) showed significantly lower activity (yield of up to 22%), but better selectivity towards vinylidene oligomers (up to 95%). This dependence of regioselectivity on the nature of the transition metal was confirmed in an experiment with ^13^C-labeled hexene: the cross-linking of an alkene in the case of a Zr catalyst occurs as successive stages of a 1,2-insertion of an olefin into M-H species, a 1,2-insertion of an alkene into M-Alkyl, and β-H elimination. In the case of Ti, the stages of 1,2-olefin insertion into M-H, 2,1-alkene insertion into M-Alkyl, and β-H elimination occur. The rate of 2,1-olefin insertion is affected by solvation, an increase in the bulkiness of the ligand and the growing chain, as well as temperature. Thus, low temperatures down to −80 °C in the case of **36a** led to the following ratio: [vinylene]/[vinylidene] = 52/48.

**Scheme 19:**
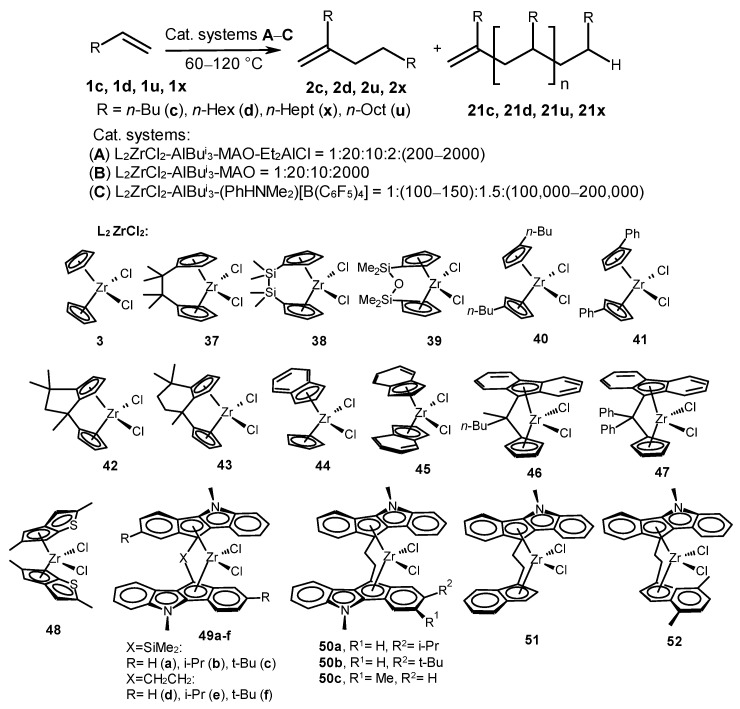
Alkene dimerization and oligomerization catalyzed by complexes **3** and **37**–**52**.

Dimers and oligomers of terminal alkenes were synthesized in catalytic systems based on various zirconocenes (**3** and **37**–**52**) with cyclopentadienyl ligands; indenyl ligands; fluorenyl ligands, including *ansa*-complexes; and heterocenes, which were activated in several steps by AlBu^i^_3_, Et_2_AlCl, and methylaluminoxane (Scheme 19) [5,40,59,60,61,62]. Cyclopentadienyl complexes, including Cp_2_ZrCl_2_ (**3**), (Me_2_C)_2_Cp_2_ZrCl_2_ (**37**), (Me_2_Si)_2_Cp_2_ZrCl_2_ (**38**), and OSiMe_2_Cp_2_ZrCl_2_ (**39**), at low Al_MAO_/Zr ratios (1–10), catalyzed the regio- and chemoselective formation of head-to-tail α-olefin dimers with yields of 82–94% and 100% alkene conversion [40,61]. The oligomers of α-olefins (1-hexene, 1-octene, and 1-decene) were obtained in the reactions, catalyzed by zirconocenes **40**, **41**, **42**, and **48** and organoaluminum cocatalysts at the following ratio: [Zr]:[AlBu^i^_3_]:[MAO]:[1-alkene] = 1:20:10:2000 [40,59,60,62]. A yield of 1-hexene dimer decreased to 40–52% and a yield of oligomers increased to 55–57% under the same conditions in the presence of the CpIndZr_2_Cl_2_ complex (**44**) [59,60]. Higher 1-hexene oligomers with Mw = 3900 Da were produced by the Ind_2_ZrCl_2_ complex (**45**) [59]. The TOF values were (1–2.4)·10^5^ h^−1^ when **46** and **47** were used in the oligomerization of alkenes **1c**, **1d**, **1u**, and **1x** [40].

**Scheme 20:**
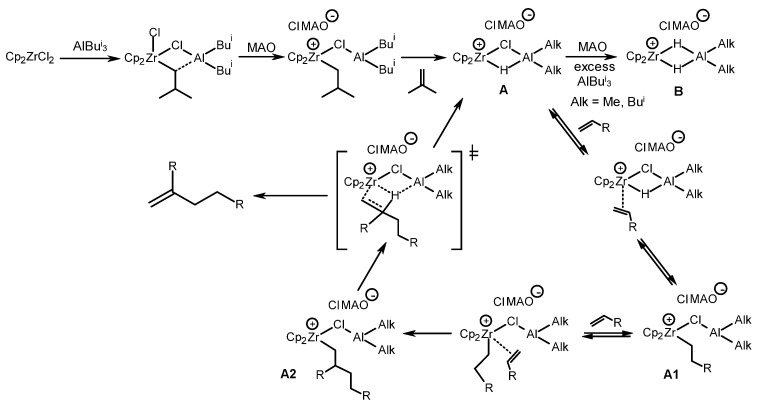
α-Olefin dimerization mechanism [59].

**Scheme 21:**
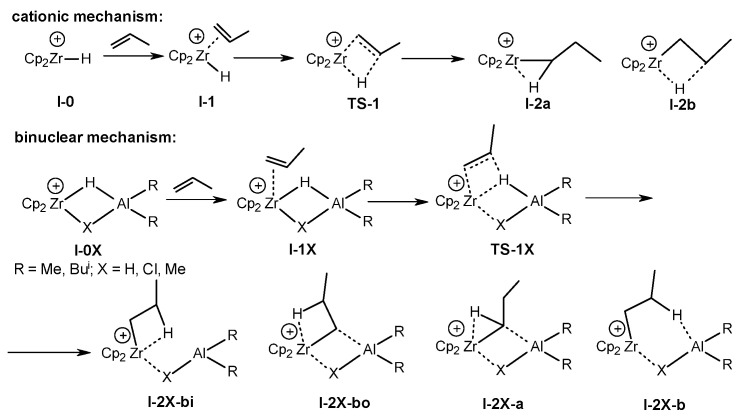
DFT modeling of the initiation stages of propene dimerization and oligomerization for cationic and binuclear mechanisms [62].

**Scheme 22:**
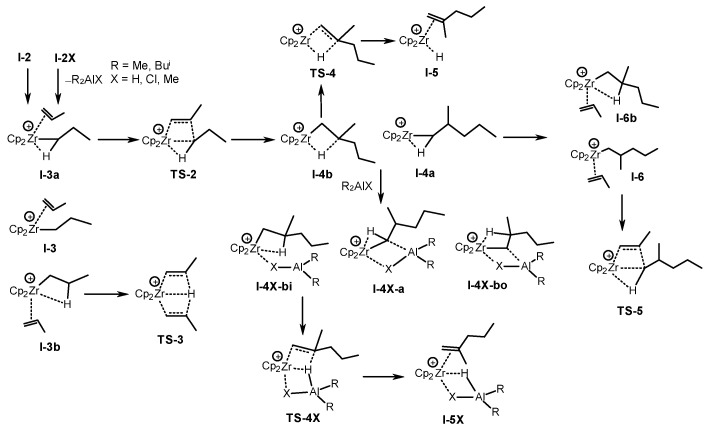
DFT modeling of propagation and termination stages of the propene dimerization and oligomerization [62].

To explain the catalytic action of the systems, the mechanisms presented in Scheme 20, Scheme 21 and Scheme 22 were suggested. For example, Zr,Al complex **A** stabilized by the ClMAO^–^ anion [63] formed in the reaction of Cp_2_ZrCl_2_ with AlBu^i^_3_ and MAO was proposed as a catalytically active center for the alkene dimerization reaction (Scheme 20) [59]. An excess of OAC (MAO or AlBu^i^_3_) increases the amount of dihydride complex **B**. Catalytically active center **A** coordinates an alkene molecule at the Zr–H bond to form alkyl complex **A1**, the further alkylation of which provides intermediate **A2**. Chlorine atoms in complex **A2** promote the process of β-H transfer to the Zr atom, rather than the coordination of the third and subsequent substrate molecules (chain growth). An alkene dimer and catalytically active center **A** are formed after β-H transfer to a Zr atom. Intermediate **B** is electrophilic and seems to be sterically accessible for α-olefin oligomerization. An increase in selectivity of the reaction observed in the dimerization after the treatment of a reaction mixture with Et_2_AlCl is probably due to the conversion of **B** to **A** (Scheme 20). The selectivity of the dimerization reaction of α-olefins, therefore, depends mainly on the ratio of catalytically active sites **A** and **B**.

The initial stages of the propene dimerization and oligomerization with the participation of Zr,Al–complexes were simulated at the DFT M-06X/DGDZVP level of theory to confirm the proposed mechanism [62]. The profiles of propene oligomerization reactions catalyzed by [Cp_2_ZrH]^+^ cation **I-0** and cationic bimetallic complexes [Cp_2_Zr(µ-H)(µ-X)AlR_2_]^+^ (X = H, Cl, and Me; R = Me and Bu^i^) **I-0X** were constructed (Scheme 21). Further, activation energies were calculated for the two reaction pathways: the formation of a vinylidene propene dimer via **TS-4** and the chain growth via **TS-5** (Scheme 22).

A difference between the mechanisms for traditional mononuclear [Cp_2_Zr-alkyl]^+^ and binuclear [Cp_2_Zr-alkyl(R_2_AlX)]^+^ species is shown (Scheme 22). Without R_2_AlX coordination, oligomerization is the favored reaction route. When X = H, highly stable β-agostic complexes **I-2X-bo** form, so the reactions slow down. If X = Cl, the main direction of the action is the formation of vinylidene dimers. The transition states of β-H elimination **TS-4X** (X = H and Cl) show a Zr-Al concerted effect. If X = Me, then there is no significant promotion in the β-H elimination process in **TS-4** [62].

It was found that the use of molecular hydrogen at a low MAO concentration leads to the results being not typical for Ziegler–Natta processes [62]. The dimerization accelerates, and the selectivity of the reaction in this pathway increases without the formation of hydrogenolysis products in the presence of hydrogen. The DFT simulation showed that the **I-2H-bo** complex can react with H_2_ without breaking an H-H bond but with a loss of β-agostic coordination. Molecular hydrogen, therefore, acts as an additional activator for the **I-2H-bo** hydride complex, which is probably an active and selective catalyst of a dimerization reaction.

Zr heterocene complexes **48** and **49a**–**f** modified with AlBu^i^_3_ and MMAO-12 were studied in the reaction of 1-decene oligomerization in molecular hydrogen at a [Zr]:[Al]:[MAO]:[1-alkene] ratio of 1:10:75:50,000 at 80–100 °C (Scheme 13) [64]. The conversion of 1-decene reached 99% in the presence of **49d** at 80 °C for 4 h, and the formation of low-viscosity oligomers was observed. As the temperature rises to 100 °C, the content of 1-decene dimer increases to 28%. Nevertheless, heterocene **49f** was shown to be the most effective catalyst for the synthesis of low-viscosity 1-decene oligomers among the studied complexes. Moreover, the catalytic system based on complex **49f** and an activator, (PhHNMe_2_)[B(C_6_F_5_)_4_], enabled us to achieve a maximum yield (63 wt%) of the most valuable trimer–tetramer fractions of alkene oligomers at a [Zr]:[Al]:[B]:[1-alkene] ratio of reagents of 1:150:1.5:200,000 in 1 atm H_2_ at 80–110 °C [64].

Unsymmetrical complexes **50a**–**c**, **51**, and **52** in the presence of AlBu^i^_3_, (PhHNMe_2_)[B(C_6_F_5_)_4_], and H_2_ (1 bar) at a [Zr]:[Al]:[B]:[1-alkene] ratio of 1:100:1.5:100,000 at 100 °C catalyzed the formation of light 1-decene oligomers with an alkene conversion rate of 86–99% [65]. A gradual decrease in the reaction temperature as 1-decene was shown to reduce the content of dimers (down to 10%) and increase the proportion of oligomers (up to 84%) in the reaction products.

**Scheme 23:**
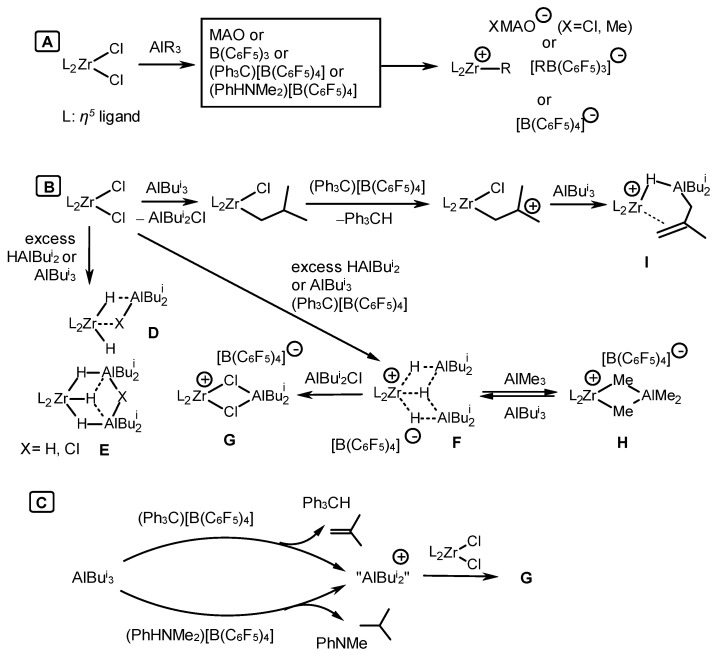
Catalytic species observed in the systems L_2_ZrCl_2_-AlBu^i^_3_(HAlBu^i^_2_)-activator [64].

The authors of [64] presented a mechanism for the activation of zirconocene complexes by isobutylalanes, arylboranes, and MAO, as depicted in Scheme 23. They noticed that the classical mechanism implies the participation of active catalytic species, such as alkyl zirconocene cations (L_2_Zr-R^+^ (L_2_ = η^5^-ligands)) stabilized by [B(C_6_F_5_)_4_]^–^, [B(C_6_F_5_)_3_R]^–^, or XMAO^–^ counterions (X = Cl and Me) (Scheme 23A). The reaction between L_2_ZrCl_2_ and AlBu^i^_3_ produces zirconocene alkyl chloride, L_2_Zr(Cl)Bu^i^. An excess of AlBu^i^_3_ or HAlBu^i^_2_ provides various neutral hydride Zr,Al complexes **D** and **E** (Scheme 23B). A cationic hydride bimetallic complex **F** is generated in the presence of perfluoroarylboranes (Scheme 23B). Under the action of excess ClAlBu^i^_2_, cation **F** transforms into dichloride Zr,Al-complex **G**, which can also be formed by the reaction between L_2_ZrCl_2_ and R_2_Al^+^. Complex **G** was isolated and characterized by NMR and an X-ray diffraction analysis [64]. Cationic hydride complex **F** belongs to the category of dormant states as well as species [L_2_Zr-(μ-Me)_2_-AlMe_2_]^+^ (**H**). Alkenyl hydride Zr-(μ-H)-Al complex **I** formed in the presence of excess AlBu^i^_3_ is considered to be potentially active towards α-olefins (Scheme 23B) [64]. However, a complex similar to **I** was shown to be inactive in alkene polymerization [66,67]. Moreover, the reaction mechanism involving metallocenes and AlBu^i^_3_ should take into account the participation of a cationic species, “AlBu^i^_2_^+^”, formed as a result of the reaction of OAC with a boron activator.

**Scheme 24:**
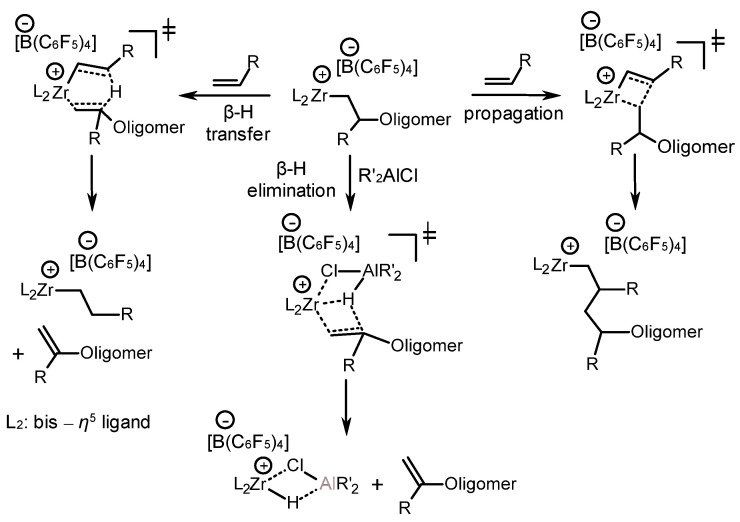
Various reaction directions in the course of alkene oligomerization [65].

Further, when considering the possible stages of alkene oligomerization (which are coordination, chain growth, and termination, involving the β-H transfer and β-H elimination stages), the authors noted [65] that in the case of heterocenes, the processes of β-H elimination apparently prevail at the final stage of the reaction, when most of the monomer is consumed, leading to the accumulation of C20 dimers in the products (Scheme 24). The β-H elimination can be facilitated by the coordination of R’_2_AlCl at the Zr center. The competing processes of chain propagation and termination are influenced by both steric and electronic factors of the η^5^-ligand. It is noted that electron-donating alkyl substituents in the ligand of the complex lead to a decrease in the electrophilicity of the Zr atom and, consequently, to a decrease in catalytic activity, for example, in the case of **49f** vs. **50a** and **50b**. Nevertheless, the lower electrophilicity of Zr (**49f**) or steric hindrances (for example, in the case of **51** or **52**) of the ligand does not promote β-H transfer, which provides higher yields of C30+ or C50+ oligomers [65].

**Scheme 25:**
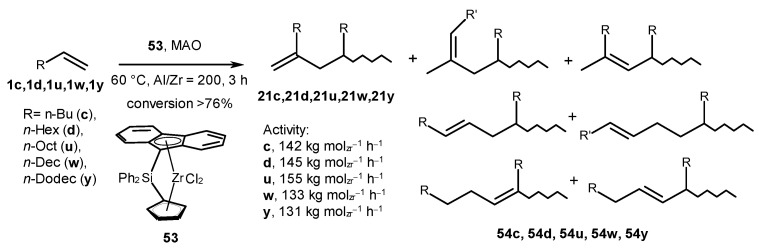
Alkene oligomers obtained in the reaction catalyzed by *ansa*-Ph_2_Si(Cp)(9-Flu)ZrCl_2_ (**53**) [68].

The alkene oligomers were obtained in the reaction catalyzed by *ansa*-Ph_2_Si(Cp)(9-Flu)ZrCl_2_ (**53**) in the presence of MAO at an Al/Zr of 200 and a temperature of 60 °C (Scheme 25) [68]. This system showed the activity to be 131–155 kg mol_Zr_^−1^ h^−1^ (PDI = 2.06–2.25). The oligomers constituted a mixture of regioisomeric products with a terminal vinylidene (**21**) and internal double bonds (**54**) according to the ^1^H and ^13^C NMR spectra. Oligomers with vinylene R′CH=CHR″ and vinylidene CH_2_=CHR′R″ groups were the major products. Oligomers containing internal disubstituted vinylene groups were formed through 2,1-insertion and β-H elimination or 2,1-insertion and rearrangement, followed by β-H elimination. An NMR analysis of the intensities of the double bond signals and saturated end groups showed the preferential chain transfer to the cocatalyst. 

**Scheme 26:**
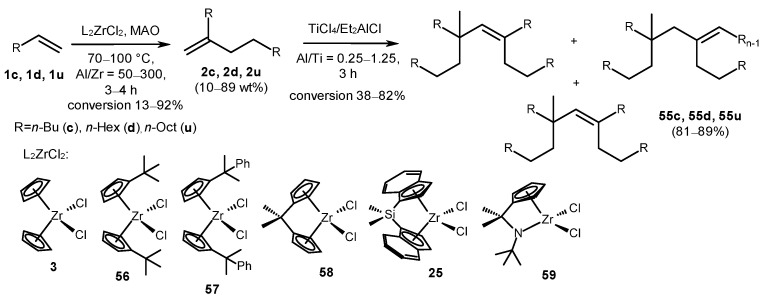
Alkene transformations into dimers and tetramers [69].

Zirconocenes (**3**, **25**, **56**–**59**) and methylaluminoxane catalyzed terminal alkene transformation into dimers **2c**, **2d**, and **2u** with yields of up to 89% at a [Zr]:[MAO]:[1-alkene] reactant ratio of 1:(50–300):(289–2600) and a temperature of 70–100 °C for 3–4 h (Scheme 26) [69]. Catalysts **25** and **59** exhibited the highest activity in the oligomerization reaction. Complex **59** demonstrated superior selectivity towards dimer formation. Dimers **2c**, **2d**, and **2u** were converted into tetramers (**55c**, **55d**, and **55u**) under the action of a TiCl_4_-Et_2_AlCl system. 

**Scheme 27:**
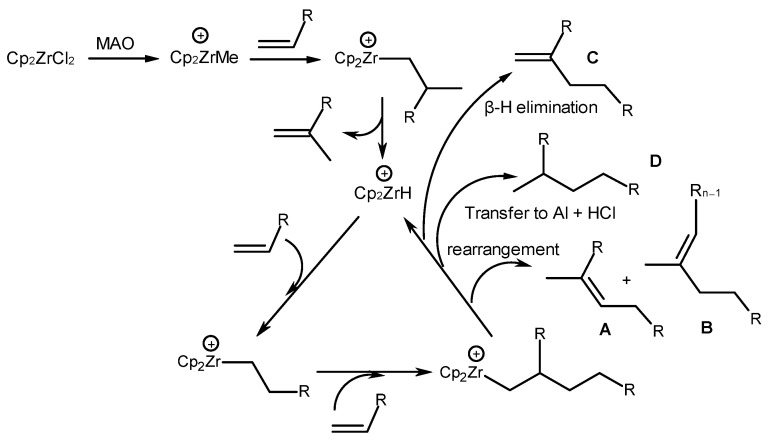
Mechanism of metallocene-catalyzed dimerization [69].

The authors proposed a mechanism for metallocene-catalyzed dimerization based on a structural analysis of alkene dimers (Scheme 27) [69]. The formation of unsaturated (structures **A**–**C**) and saturated products (structure **D**) occurred due to the β-H elimination at cationic metal alkyl centers and chain transfer to a non-transition metal atom (Al), respectively [69]. The vinylidene group (-C=CH_2_) (structure **C**) was generated via a 1,2-coordination of an alkene with a [Cp_2_ZrH]^+^ cation and the subsequent β-H elimination of the product. Alkene 1,2-coordination, cation rearrangement, and β-H elimination produce structures **A** and **B** with trisubstituted vinyl groups (-C=C(CH_3_)-).

**Scheme 28:**
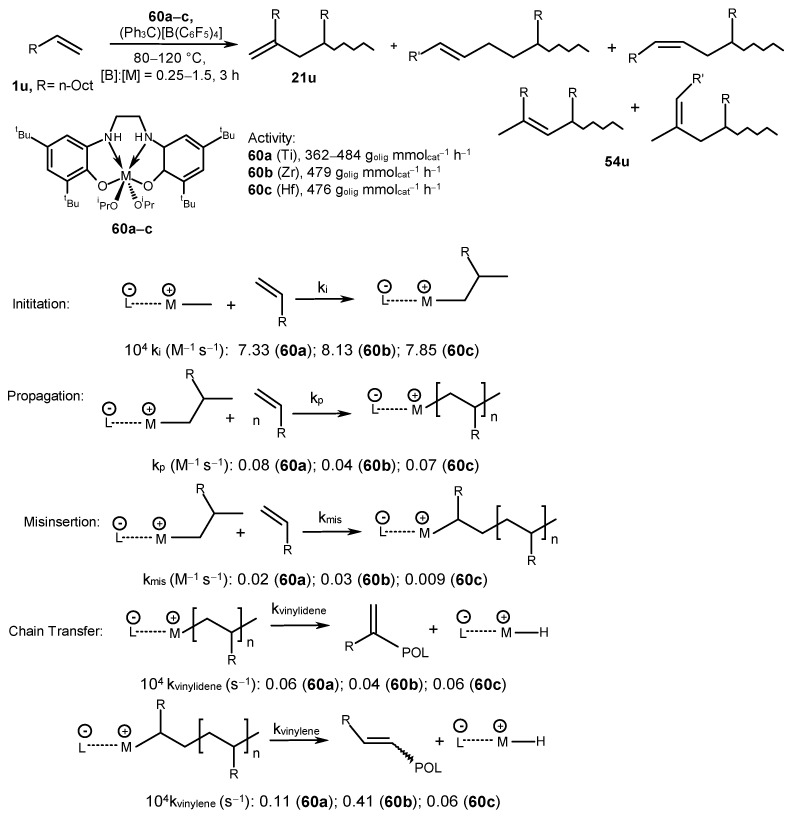
1-Decene oligomerization, catalyzed by complexes **60a**–**c**, and kinetic parameters of the reaction [70].

1-Decene was transformed into oligomers under the action of post-metallocene complexes [M{2,2′-(OC_6_H_2_-4,6-^t^Bu_2_)_2_NHC_2_H_4_NH}(O^i^Pr)_2_] (**60a**–**c**) (M = Ti (**a**), Zr (**b**), and Hf (**c**)) and the (Ph_3_C)[B(C_6_F_5_)_4_] activator at a [B]:[M] ratio of 1:(0.25–1.5) at 80–120 °C [70] (Scheme 28). The activity of the catalytic system was quantified as 362–484 g_oligomer_ mmol_cat_^−1^ h^−1^. The resulting oligomers were characterized by the tacticity values (mm + rr) of 88.5% (Ti), 87.3% (Zr), and 86.8% (Hf); the molecular weight (M_N_ = 445–608 g mol^−1^); and the PDI value (1.13–1.30). The resulting oligomers differed in structure and contained vinylidene fragments (CH_2_=CRR′ (**21u**, δ_H_ 4.7–4.8 ppm)), vinyl fragments (CH_2_=CHR (**54u**, δ_H_ 4.9 and 5.6 ppm)), trisubstituted vinylene groups (RCH=CR′R″ (**54u**, δ_H_ 5.2 ppm)), and disubstituted vinylene groups (RCH=CHR′ (**54u**; δ_H_ 5.3–5.5 ppm)).

The monomer consumption, the number of active sites, and the number of unsaturated end groups during the oligomerization reaction were evaluated for each catalytic system in the course of the study of the kinetics of 1-decene oligomerization reaction catalyzed by **60a**–**c** (Scheme 28) [70]. An initiation rate constant (k_i_) in the presence of complex **60b** appeared to be higher than those of **60a** and **60c** (Scheme 28). The k_i_ value was inversely related to the molecular weight of an oligomeric product. A catalyst with a high k_i_, when the number of active centers is high, leads to low-molecular-weight oligomers. The Ti-based catalytic system exhibited a higher chain propagation rate compared to those of the Zr- and Hf-based systems. Moreover, the reaction initiation stage was found to be slower in comparison to the chain propagation. A decrease in the chain growth constants k_p_ in the Ti > Hf > Zr series was probably due to the electronic nature of metal centers. The rate of formation of a vinylidene product did not depend on the concentration of 1-decene, whereas the rate of formation of a product with an internal double bond was of the first order relative to the monomer concentration. The k_vinylidene_ and k_vinylene_ values were calculated from the initiation rate constants, k_i_, where k_vinylene_ > k_vinylidene_ by a factor of 2–10. The degree of catalyst involvement in the reaction was 40–60%. The misinsertion stage was slower than the propagation stage for all studied catalysts. The chain termination process runs via the chain β-H transfer to a monomer and the β-H elimination reaction (Scheme 28) [70].

**Scheme 29:**
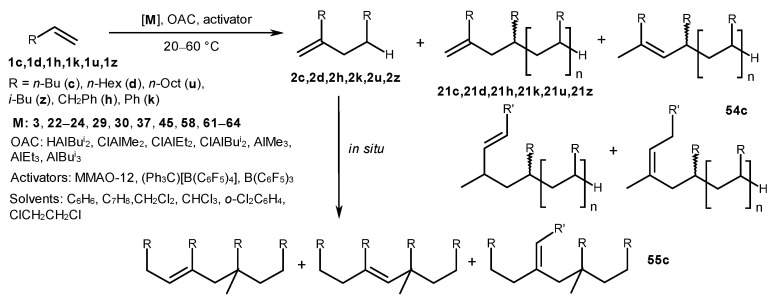

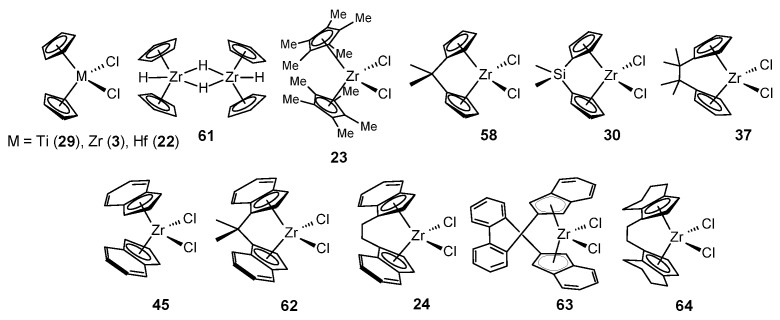
Alkene transformations upon the action of metallocene–OAC–activator catalytic systems [71,72,73,74].

A study of the activity and chemoselectivity of η*^5^*-metal complexes **3**, **22**–**24**, **29**, **30**, **37**, **45**, **58**, and **61**–**64** in the presence of various OACs (HAlBu^i^_2_, ClAlMe_2_, ClAlEt_2_, ClAlBu^i^_2_, AlMe_3_, AlEt_3_, and AlBu^i^_3_) and activators (MMAO-12, (Ph_3_C)[B(C_6_F_5_)_4_], and B(C_6_F_5_)_3_) in alkene dimerization and oligomerization showed that either HAlBu^i^_2_ or AlBu^i^_3_ at certain ratios ensure the selectivity of the reaction towards dimerization in comparison with AlMe_3_ or AlEt_3_ (Scheme 29) [71]. Moreover, Cp_2_ZrCl_2_-(AlBu^i^_3_ or HAlBu^i^_2_) or [Cp_2_ZrH_2_]_2_-ClAlR_2_ (R = Me, Et, Bu^i^) systems produced predominantly head-to-tail dimers (**2c**,**d**,**h**,**k,u**,**z**) in the presence of MMAO-12 or B(C_6_F_5_)_3_ activators at the [Zr]:[Al]:[MMAO-12]:[1-alkene] ratio of 1:3:30:(50–1000) or the [Zr]:[Al]:[B]:[alkene] ratio of 4:16:1:1000, correspondingly, at 20–60 °C for 5–180 min in toluene with a yield of up to 98% (**2c**, 98%; **2d**, 91%; **2u**, 87%; **2z**, 95%; **2h**, 61%; **2k**, 58%) (Scheme 29) [71,72].

The use of chlorinated solvents (CH_2_Cl_2_ and CHCl_3_) in the Cp_2_ZrY_2_-YAlBu^i^_2_ (Y = H, Cl) systems’ activator (MMAO-12, (Ph_3_C)[B(C_6_F_5_)_4_]) accelerated the reaction and increased the yield of dimeric products [73]. Under these conditions, the dimers obtained in the first minutes were substrates for the subsequent dimerization and formation of tetramer **55** with yields of up to 79%. Adding an ionic-type cocatalyst, (Ph_3_C)[B(C_6_F_5_)_4_], to either the Cp_2_ZrCl_2_-HAlBu^i^_2_ or [Cp_2_ZrH_2_]_2_-ClAlBu^i^_2_ catalytic systems typically resulted in the formation of oligomeric products [72]. Replacing the transition metal atom from Zr to Ti or Hf under the same conditions led to a decrease in activity and selectivity towards dimers [73].

A study on the influence of the ligand structure on the activity and chemoselectivity of the system L_2_ZrCl_2_-HAlBu^i^_2_-MMAO-12 revealed that dimerization occurs with the participation of Zr complexes with sterically unhindered ligands (L = Cp, *ansa*-Me_2_CCp_2_, *ansa*-(Me_2_C)_2_Cp_2_, and *ansa*-Me_2_SiCp_2_) [74]. Zirconocenes with bulky cyclopentadienyl (L = C_5_Me_5_ and *rac*-H_4_C_2_[THInd]_2_) or electron-withdrawing indenyl (L = Ind, Me_2_CInd_2_, H_4_C_2_[Ind]_2_ and BIPh(Ind)_2_) substituents in the presence of HAlBu^i^_2_ and MMAO-12 or (Ph_3_C)[B(C_6_F_5_)_4_] activators predominantly yielded 1-hexene oligomers, which is consistent with the data in Ref. [60]. The assessment of the stereoselectivity of the reaction using ^13^C NMR spectroscopy showed a dependence of this parameter on the *π*-ligand environment of the metal and the type of activator [74]. Catalysts with indenyl ligands **45**, **62**, and **24** were found to be the most stereoselective, demonstrating isotacticity levels of 67%, 93%, and 71%, respectively. An oligomer with an isotacticity level of 67% was obtained under the action of complex **45** in the presence of MMAO-12, whereas (Ph_3_C)[B(C_6_F_5_)_4_] led to an atactic product. The opposite situation was observed for complex **62** with *ansa*-bridged ligands: the highest stereoselectivity was achieved in the presence of (Ph_3_C)[B(C_6_F_5_)_4_].

**Scheme 30:**
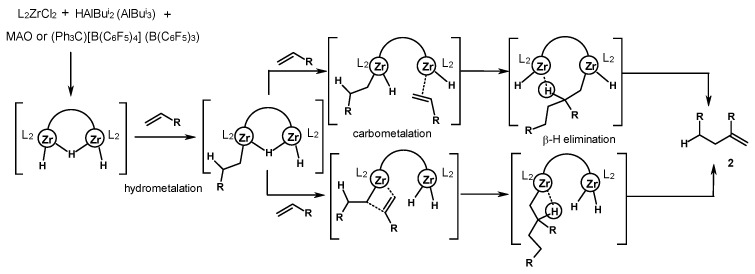
Probable mechanism of alkene dimerization [72].

These facts indicate that a cocatalyst has a significant influence on the stereoregulation process during the alkene coordination through catalytically active centers. As a result, the data on the structure and reactivity of possible intermediates [71,72,74,75], the high selectivity of a reaction towards the dimerization, and completely different rates of oligomerization and dimerization processes allow us to propose a mechanism (Scheme 30). The mechanism implies the involvement of bis-zirconium hydride structures as precursors of dimerization reaction active sites. At the first stage of the reaction, the hydrometalation of alkenes proceeds with the participation of one of the zirconium centers. The introduction of the second alkene molecule, the carbometalation stage, and the β-H elimination stage can also proceed in concert with the involvement of two zirconium atoms. Finally, the dimerization product (**2**) and the starting bis-zirconium complex are formed. Examples of such bimetallic catalysis are known for the polymerization of alkenes in the presence of subgroup 4 metal complexes [76], as well as ethylene tetramerization reactions on chromium catalysts [77,78,79].

Thus, the literature provides extensive information on the dimerization and oligomerization of alkenes under the action of homogeneous catalytic systems based on metallocenes and post-metallocenes. Typically, these works emphasize the key roles of metal hydride intermediates as active species. Therefore, the study of the structure and reactivity of hydride complexes of transition metals is a relevant task to develop models of reaction mechanisms.

## 3. Structure of Catalytically Active Centers

### 3.1. Reactions of Organoaluminum Compounds with Activators and Metal Complexes

**Scheme 31:**
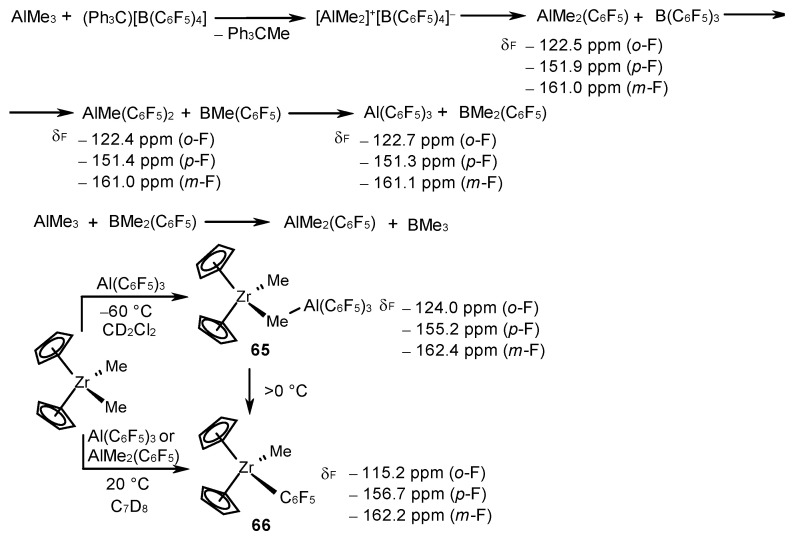
Reaction of AlMe_3_ with (Ph_3_C)[B(C_6_F_5_)_4_] and Cp_2_ZrMe_2_ [80].

**Scheme 32:**
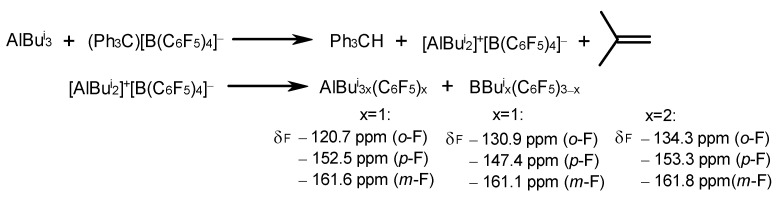
Reaction of AlBu^i^_3_ with (Ph_3_C)[B(C_6_F_5_)_4_] [80].

Many researchers noted that the formation of catalytically active centers for the oligo- and polymerization of alkenes is preceded by the interaction of the activator with organoaluminum compounds. For example, in the reaction of AlR_3_ (R = Me, Et, and Bu^i^) with a B-containing activator upon heating and different Al/B ratios, the formation of a mixture of AlR_3–x_(C_6_F_5_)_x_ derivatives was found (Scheme 31 and Scheme 32) [80]. The NMR monitoring of the reaction of AlMe_3_ with (Ph_3_C)[B(C_6_F_5_)_4_] in d_8_-toluene at a temperature of 60 °C for 4.5 h showed that MeCPh_3_ (δ_H_ 0.74 ppm) and BMe_3_ (δ_B_ 86.8 ppm) were formed. It is assumed that the interaction between AlMe_3_ and (Ph_3_C)[B(C_6_F_5_)_4_] first gives the intermediate [AlMe_2_]^+^[B(C_6_F_5_)_4_]^–^, which immediately decomposes to AlMe_2_(C_6_F_5_) and B(C_6_F_5_)_3_ (Scheme 31). The transformation of [B(C_6_F_5_)_4_]^-^ is started due to the generation of the highly electrophilic “[AlR_2_]^+^” cation. Over time, the replacement of the Me group in the OAC molecule by C_6_F_5_ occurs to provide the final products, Al(C_6_F_5_)_3_ and BMe_3_. Moreover, neutral B(C_6_F_5_)_3_ also participates in ligand exchange with AlMe_3_. The organoaluminum products of intermolecular exchange differed in the values of the ^19^F NMR chemical shifts δ_F_ presented in Scheme 31. The further interaction of Al(C_6_F_5_)_3_ with Cp_2_ZrMe_2_ at −60 °C in CD_2_Cl_2_ provided [Cp_2_ZrMe(µ-Me)Al(C_6_F_5_)_3_] (**65**). In the ^1^H NMR spectrum of compound **65**, singlet signals of protons were observed: a Cp ring at δ_H_ 6.44 ppm and Zr-Me and Zr-Me-Al groups at δ_H_ 0.51 and −0.26 ppm, respectively. The reaction of Cp_2_ZrMe_2_ with AlMe_2_(C_6_F_5_) or Al(C_6_F_5_)_3_ (at a Zr:Al ratio of 1:1) in d_8_-toluene at room temperature provided a yellow complex, [Cp_2_ZrMe(C_6_F_5_)] (**66**), and the ^1^H NMR spectrum exhibited characteristic signals of both Cp and Me groups at δ_H_ 5.66 and 0.31 ppm, correspondingly [80].

The reaction of AlBu^i^_3_ with (Ph_3_C)[B(C_6_F_5_)_4_] was accompanied by the elimination of isobutene, Ph_3_CH, and the generation of anunstable ionic pair, [AlBu^i^_2_]^+^[B(C_6_F_5_)_4_]^–^, which also decomposed to AlBu^i^_3–x_(C_6_F_5_)_x_ and BBu^i^_x_(C_6_F_5_)_3–x_ (Scheme 32) [80]. 

**Scheme 33:**
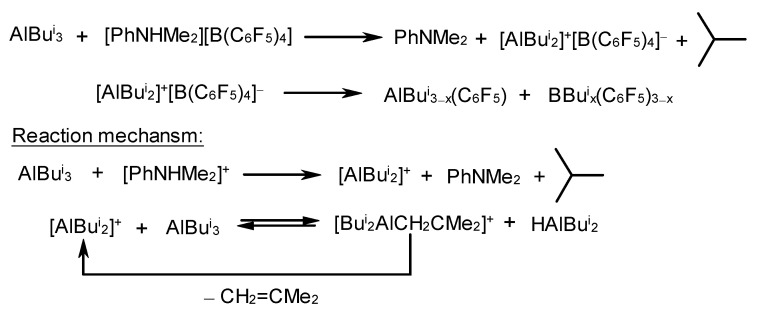
Reaction of AlBu^i^_3_ with an activator, (PhNHMe_2_)[B(C_6_F_5_)_4_] [67].

A similar reaction of AlBu^i^_3_ with an activator, (PhNHMe_2_)[B(C_6_F_5_)_4_], produced AlBu^i^_3–x_(C_6_F_5_)_x_, isobutane, and PhNMe_2_, which was assumed to proceed through the formation of an ionic pair, [AlBu^i^_2_]^+^[B(C_6_F_5_)_4_]^–^, according to Scheme 33 [67]. Then, the ionic pair, [AlBu^i^_2_]^+^[B(C_6_F_5_)_4_]^–^, transformed into AlBu^i^_3−x_(C_6_F_5_)_x_ and BBu^i^_x_(C_6_F_5_)_3−x_. The reaction of AlBu^i^_3_ with an activator, apparently, yields the “[AlBu^i^_2_]^+^” species, which further reacts with excess AlBu^i^_3_, producing HAlBu^i^_2_ and [Bu^i^_2_AlCH_2_CMe_2_]^+^. The latter, upon losing isobutylene, regenerates the “[AlBu^i^_2_]^+^” cation.

**Scheme 34:**
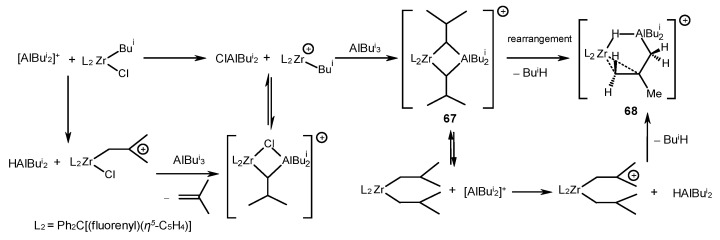
Proposed mechanism of complex **68** formation [67].

Accumulating in the system, the [AlBu^i^_2_]^+^ cation removes a chlorine atom or a β-H from the Ph_2_C(CpFlu)ZrClBu^i^ complex to provide [Ph_2_C(CpFlu)ZrBu^i^]^+^ (Scheme 34). Subsequently, upon reacting with an excess of AlBu^i^_3_, it yields a binuclear complex, [Ph_2_C(CpFlu)ZrBu^i^·AlBu^i^_3_]^+^ (**67**), which further provides metallocycle [Ph_2_C(CpFlu)Zr(*µ*-H)(*µ*-C_4_H_7_)AlBu^i^_2_]^+^ (**68**) as a result of isobutane elimination [67].

**Scheme 35:**
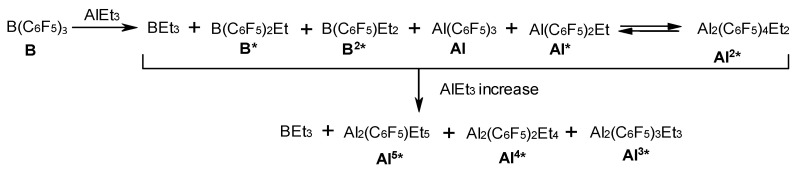
Reaction of B(C_6_F_5_)_3_ with AlEt_3_ [81].

In the course of NMR monitoring of the reaction of B(C_6_F_5_)_3_ with AlEt_3_ in CD_2_Cl_2_, Al(C_6_F_5_)_3–n_Et_n_ monomers and Al_2_(C_6_F_5_)_6–n_Et_n_ dimers were observed [81]. Depending on the ratio of B(C_6_F_5_)_3_ and OAC, the Al(C_6_F_5_)_3_ → Al(C_6_F_5_)_2_Et ↔ Al_2_(C_6_F_5_)_4_Et_2_ → Al_2_(C_6_F_5_)_3_Et_3_ → Al_2_(C_6_F_5_)_2_Et_4_ → Al_2_(C_6_F_5_)Et_5_ compounds formed, which were clearly distinguished in the ^19^F NMR spectra by the signal of the *p*-F substituent in the benzene ring. For example, at a [B(C_6_F_5_)_3_]:[AlEt_3_] ratio of 1:9, the Al_2_(C_6_F_5_)_2_Et_4_ (Al^4^*) and Al_2_(C_6_F_5_)Et_5_ (Al^5^*) dimers, together with a monomer, Al(C_6_F_5_)_2_Et, were identified (Scheme 35). 

**Scheme 36:**
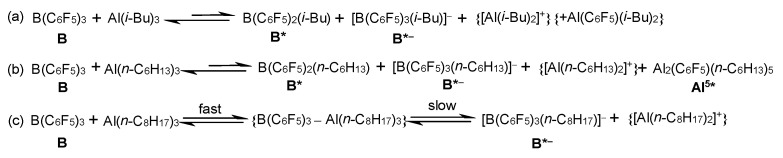
Reaction of B(C_6_F_5_)_3_ with AlR_3_ (a) R = i-Bu, (b) R = n-C_6_H_13_, (c) R = n-C_8_H_17_) [81].

Higher organoaluminum compounds AlR_3_ (R = *i*-Bu and *n*-C_6_H_13_) were also capable of participating in an exchange reaction with B(C_6_F_5_)_3_ [81]. The starting arylborane and its anion, [B(C_6_F_5_)_3_R]^–^, were in equilibrium at an equimolar ratio (Scheme 36). A large excess of AlR_3_ shifts the equilibrium towards the exchange products—BR_3_ and Al(C_6_F_5_)R_2_ (R = Bu^i^ and n-C_6_H_13_). The signals of B(C_6_F_5_)_3_ (**B**), B(C_6_F_5_)_2_(Bu^i^) (B*), and [B(C_6_F_5_)_3_(Bu^i^)]^–^ (B*^–^) were detected in the ^19^F NMR spectrum of the reaction mixture of AlBu^i^_3_ with B(C_6_F_5_)_3_. There was almost no alkyl exchange with B(C_6_F_5_)_3_ in the case of higher trialkylalane Al(n-C_8_H_17_)_3_ [81].

The MAO cocatalyst also exchanges with OACs similar to B-containing activators. An NMR study, for example, showed that Bu^i^_2_Al(*µ*-Me)_2_AlBu^i^_2_ dimers and (AlMe_(1+2x−y)_Bu^i^_y_O(_1−x_))_n_ clusters, whose methyl and methylene protons gave the signals at δ_H_ 1.12 and 0.34 ppm, respectively, formed in a system, (SBI)ZrCl_2_-MAO-AlBu^i^_3_ [82]. These clusters (types I and II) contain aluminum centers with higher Lewis acidity levels compared to the corresponding clusters in the original MAO, judging by the EPR signals observed in these solutions upon the addition of TEMPO (2,2,6,6-tetramethylpiperidin-1-yl)oxyl). The clusters were characterized by hyperfine structure constants, *a*_Al_ = 1.0 ± 0.1 (I) and 1.9 ± 0.1 (II) G. When AlBu^i^_3_ was added to MAO, the constant of type II Al centers increased (*a*_Al_ = 4.0–4.5 G). As a result, ion pairs of the [(SBI)ZrMe]^+^[Me-(MAO-TIBA)]^–^ type were detected in the catalytic system ((SBI)ZrCl_2_-MAO-AlBu^i^_3_). 

**Scheme 37:**
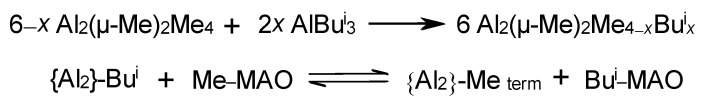
Reaction of MAO with aluminum alkyls [83].

Further, it was shown that the addition of AlBu^i^_3_ to the solutions containing methylaluminoxane (MAO) and (SBI)ZrCl_2_ provides Al_2_(*µ*-Me)_2_Me(_4−x_)Bu^i^_x_ dimers (Scheme 37) [83]. The broadened signals in the ^1^H NMR spectra at δ_H_ 0.35, 1.10, and 2.00 ppm were assigned to Bu^i^ groups bound to MAO clusters. It was assumed that Bu^i^-MAO led to the transformation of a [(SBI)Zr(μ-Me)_2_AlMe_2_]^+^ cationic adduct into [(SBI)Zr(μ-Me)_2_AlMeBu^i^]^+^ and [(SBI)Zr(μ-Me)_2_AlBu_2_^i^]^+^. These compounds were unstable and subsequently transformed into zirconocene hydrides with isobutene elimination. MAO and AlBu^i^_3_, therefore, are exchanged actively by alkyl groups to form dialuminum derivatives and mixed aluminoxanes (Scheme 37).

An NMR study of a reaction mixture (SBI)ZrCl_2_-MAO-HAlBu^i^_2_ showed that MAO exchange with HAlBu^i^_2_ provided H-substituted aluminoxane (Equation (1)) [84]. In the ^1^H NMR spectrum of the mixture, a signal of an H atom at δ_H_ 3.75 ppm was observed at relatively high ratios of [HAlBu^i^_2_]:[Zr] > 20, alongside the signals of the Ind ligands at δ_H_ 5.54 and 6.51 ppm. The signal was attributed to alkylaluminum dimers with a hydride bridge, R_2_Al(µ-R)(µ-H)AlR_2_ (R = Me or Bu^i^). The signals of Bu^i^ groups of Al_2_(µ-Me)_2_Me_4–x_Bu^i^_x_ mixed dimers were observed at δ_H_ 1.86 ppm at low [HAlBu^i^_2_]:[Zr] ratios (<20). Two broadened signals at δ_H_ 3.60 and 4.10 ppm were assigned to hydride derivatives of MAO (H-MAO):

HAlBu^i^_2_ + Me-MAO → ½ Al_2_(µ-Me)_2_Bu^i^_4_ + H-MAO (1)

**Scheme 38:**
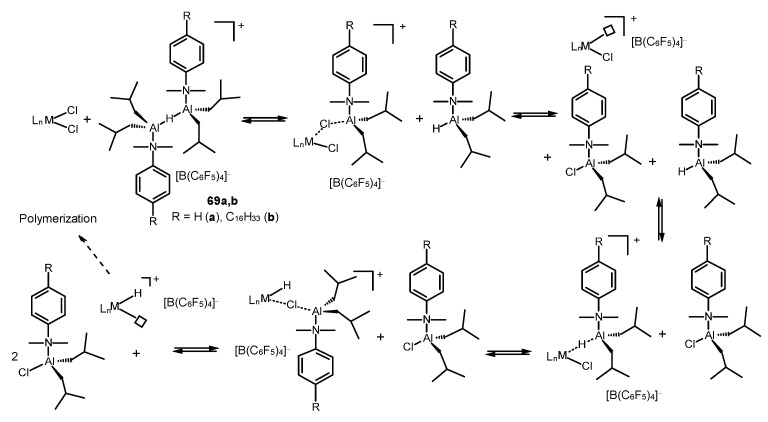
The activation of transition metal complexes in the L_n_MCl_2_-AlBu^i^_3_-[ArNMe_2_H][B(C_6_F_5_)_4_] or (Ph_3_C)[B(C_6_F_5_)_4_] systems [85].

The activation of transition metal complexes in the L_n_MCl_2_-AlBu^i^_3_-[PhNMe_2_H][B(C_6_F_5_)_4_] or (Ph_3_C)[B(C_6_F_5_)_4_] systems occurs through the “AlBu^i^_2_^+^” cation, which is generated by the rapid reaction between borate salts and AlBu^i^_3_ (Scheme 38) [85]. An excess of AlBu^i^_3_ in the system alkylates the [L_n_M-Cl]^+^ cation and provides [L_n_MBu^i^]^+^[B(C_6_F_5_)_4_]^–^ species. Moreover, the reaction of AlBu^i^_3_ with NMe_2_Ph in the presence of (Ph_3_C)[B(C_6_F_5_)_4_] produces an ionic compound, {[Bu^i^_2_(PhNMe_2_)Al]_2_(µ-H)}^+^[B(C_6_F_5_)_4_]^–^ (**69a**), which can act as an activator of the catalytic olefin polymerization reaction [85]. The ^1^H NMR spectrum of OAC **69a** exhibits singlet signals of hydride bridging atoms of the Al-H-Al bond at δ_H_ 2.86 ppm. The reaction mechanism involves the removal of the Cl atom from the initial metallocene by the [AlBu^i^_2_(NMe_2_Ph)]^+^ cation and the subsequent Cl-H exchange between [L_n_M-Cl]^+^ and the resulting HAl(NMe_2_Ph)Bu^i^_2_ to provide catalytically active hydride centers, such as [L_n_M-H]^+^ (Scheme 38). As a result, a new activator, {[Bu^i^_2_(PhNMe_2_)Al]_2_(*µ*-H)}^+^[B(C_6_F_5_)_4_]^−^ (**69a**), has been proposed, which ensures the high catalytic activity of the entire system in olefin polymerization reactions [85].

As a continuation of this research, a new cocatalyst {[(RPhNMe_2_)AlBu^i^_2_]_2_(µ-H)}^+^[B(C_6_F_5_)_4_]^−^ **69b** (R = C_16_H_33_) was synthesized based on prototype **69a** (Scheme 38). In contrast to its counterpart (**69a**), cocatalyst **69b** is highly soluble in aliphatic hydrocarbons [86]. The broadened singlet signal of protons of the Al-H-Al bridge was observed at δ_H_ 2.91 ppm in the ^1^H NMR spectrum of structure **69b**. The addition of activator **69b** to the *rac*-Me_2_Si(2,6-(CH_3_)2-4-Ph-1-Ind)_2_ZrCl_2_ catalyst in the presence of AlBu^i^_3_ enabled the copolymerization of ethylene and 1-hexene at P_C2H4_ = 12 bar and 100 °C with a productivity of (0.42–0.61)·10^6^ kg mol_cat_^−1^ h^−1^. 

Thus, organoboron or aluminum activators undergo exchange reactions with aluminum alkyls or aluminum hydrides, leading to the formation of reactive species involved in the generation of catalytically active centers that initiate alkene oligo- and polymerization.

### 3.2. Structure and Reactivity of Zr,Al–Hydride and Zr,B–Hydride Complexes

The complexes of M,Al or M,B with hydride obtained in the reactions of metallocenes with organoboron or aluminum compounds attracted great attention due to their ability to function as highly active reagents or catalytically active centers of various reactions. Numerous research teams, therefore, synthesized and structurally identified the metallocene hydrides using spectral (NMR and IR) and X-ray diffraction methods.

**Scheme 39:**
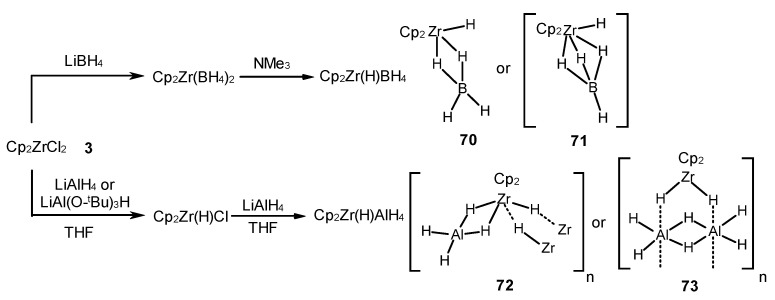
Synthesis of Zr,B– (**70**,**71**) and Zr,Al–hydride complexes (**72**,**73**).

For the first time, the synthesis of Zr,B–hydride (**70**,**71**) and Zr,Al–hydride complexes (**72**,**73**) was carried out by the reaction of Cp**_2_**ZrCl**_2_** with LiBH**_4_** [87,88] or LiAlH**_4_** [89], respectively, at room temperature (Scheme 39). The ^1^H NMR spectrum of complexes **74** and **75** exhibited a signal of Cp rings at δ_H_ 5.70 ppm and a quartet signal of four protons of the tetrahydroborate group at δ_H_ −0.20 ppm [88]. The IR spectra of complexes **76** and **77** contained absorption bands at 1425 cm^−1^ (Zr-H-Zr bond) and two bands at 1790 and 1700 cm^−1^ (AlH_4_ bond).

**Scheme 40:**
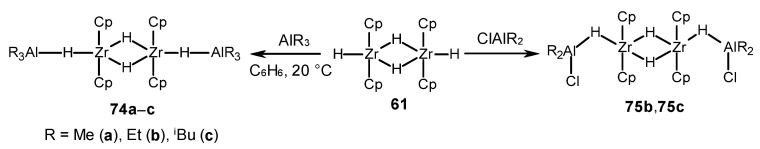
Reaction of [Cp_2_ZrH_2_]_2_ (**61**) with AlR_3_ or ClAlR_2_.

The reaction of [Cp_2_ZrH_2_]_2_ (**61**) with AlR_3_ provided complexes **74a**–**c** (Scheme 40) [90,91,92], whose ^1^H NMR spectra exhibited triplet signals of bridging hydride atoms at δ_H_ −1.23–−0.92 ppm (Zr-H-Al bond), and −2.92–−2.74 ppm (Zr-H-Zr bond). Complexes **75b** and **75c** were obtained by the reaction of **61** with dialkylchloroalanes (Scheme 40) [93]. Complexes **75b** and **75c** were characterized by a broadened triplet at δ_H_ −2.68–−2.57 ppm (Zr-H-Zr) and a broadened singlet at δ_H_ −1.73–−1.52 ppm (Zr-H-Al).

**Scheme 41:**
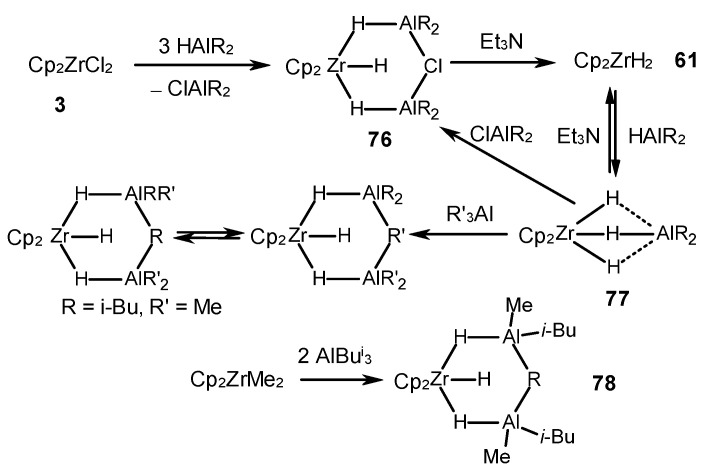
Hydride Zr,Al−complexes observed in the Cp_2_ZrCl_2_-HAlBu^i^_2_ and Cp_2_ZrMe_2_-AlBu^i^_3_ systems [94,95].

Trihydride Zr,Al complexes **76** and **77** were observed in the reaction of Cp_2_ZrCl_2_ (**3**) with 3 eq. of HAlBu^i^_2_ (Scheme 41) [94]. The ^1^H NMR spectrum of complex **77** exhibited signals corresponding to Zr-H-Al bridging hydride atoms at δ_H_ −0.28 and −2.03 ppm. Complex **76** displayed doublet and triplet signals for protons at δ_H_ −2.03 ppm (Zr-H-Al) and −0.90 ppm (Zr-H), respectively. Structure **78**, similar to complex **76**, was formed upon the interaction of Cp_2_ZrMe_2_ and AlBu^i^_3_ [95]. The ^1^H NMR spectra of the complex also featured broad singlet signals for bridging hydride atoms at δ_H_ −2.23 and −1.75 ppm, and a triplet signal for the Zr-H bond at δ_H_ −1.22 ppm.

**Scheme 42:**
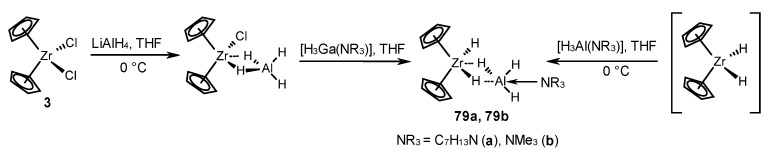
Synthesis of hydride complexes [Cp_2_Zr(H)(µ-H)_2_AlH_2_(L)] (L = C_7_H_13_N; NMe_3_) **79a** and **79b**.

Hydride complexes [Cp_2_Zr(H)(µ-H)_2_AlH_2_(L)] (L = C_7_H_13_N, NMe_3_) **79a** and **79b** were obtained in a 50% yield in the Cp_2_ZrCl_2_-LiAlH_4_ system in THF at 0 °C after the addition of H_3_GaL (Scheme 42) [96]. Complexes **79a** and **79b** can be also synthesized by the reaction of Cp_2_ZrH_2_ with [H_3_AlNR_3_]. The IR spectrum of complex **79a** showed absorption bands at 1736 and 1544 cm^−1^ (Zr-H), and for complex **79b**, bands were observed at 1768 and 1556 cm^−1^.

**Scheme 43:**
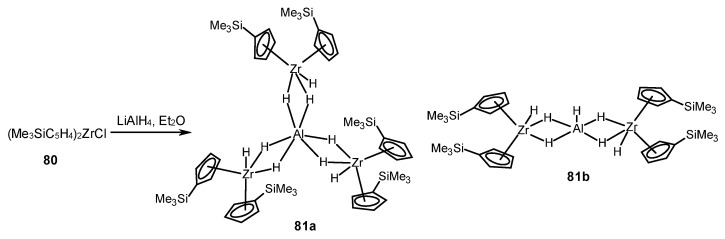
Synthesis and structure of hydride complexes **81a** and **81b**.

The reaction of (Me_3_SiCp)_2_ZrCl_2_ (**80**) with 2 eq. of LiAlH_4_ in ether at room temperature yielded hydride complexes **81a** and **81b** (Scheme 43) [97]. The broadened signals of bridging hydrides of a Zr-H-Al bond at δ_H_ 2.06 ppm and broadened signals of terminal hydrides at δ_H_ −2.57 and −2.05 ppm were detected in the ^1^H NMR spectrum of compound **81a**. According to X-ray diffraction data, compound **81** crystallizes from the solution as two structures, **81a** and **81b**. Similar structures were also obtained for Ti (III) [98,99,100].

**Scheme 44:**
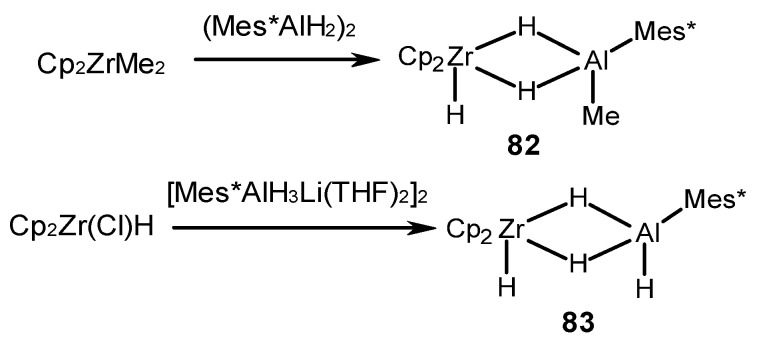
Synthesis of complexes Cp_2_Zr(H)(μ^2^-H)_2_Al(Me)Mes* (**82**) and Cp_2_(H)Zr(μ^2^-H)_2_Al(H)Mes* (**83**).

The reaction of Cp_2_ZrMe_2_ (**4**) with (Mes*AlH)_2_ (Mes* = C_6_H_2_-2,4,6-Bu^t^_3_) led to the Cp_2_Zr(H)(μ^2^-H)_2_Al(Me)Mes* complex (**82**), whereas the Cp_2_(H)Zr(μ^2^-H)_2_Al(H)Mes* complex (**83**) was formed as a result of an interaction between Cp_2_Zr(Cl)H and [Mes*AlH_3_Li(THF)_2_]_2_ (Scheme 44) [101]. The structures of **82** and **83** were elucidated using an XRD analysis, NMR, and IR spectroscopy (Scheme 44). The ^1^H NMR spectrum of complex **82** showed a doublet and singlet at δ_H_ −1.83 and −2.63 ppm (Zr-H-Al), a singlet at δ_H_ 0.02 (Al-Me), as well as a doublet of doublets at δ_H_ 2.52 ppm (Zr-H_t_, *^2^J*_HH_ = 9.0 and 5.7 Hz). Complex **83** was characterized by the broadened singlets of bridging hydrogen atoms at δ_H_ −1.99 and −2.81 ppm and a doublet of doublets at δ_H_ 2.57 ppm (*^2^J*_HH_ = 6.6 Hz, Zr-H_t_).

**Scheme 45:**
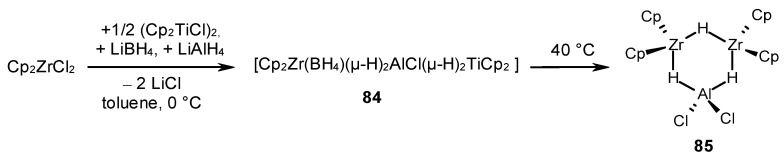
Synthesis of trinuclear heterometallic complex **85**.

A trinuclear heterometallic complex, Cp_2_Zr(X′)(*µ*-H)_2_Al(X)(*µ*-H)_2_TiCp_2_ (X, X′ = Cl, H, BH_4_) (**84**) (Scheme 45), was synthesized by the reaction of Cp_2_ZrCl_2_ and ½ (Cp_2_TiCl)_2_ with LiBH_4_ and LiAlH_4_ in toluene at 0 °C [102,103,104]. Complex **84**, upon heating to 40 °C, transformed into **85**, in which metal atoms were bound by hydride bridges, forming a six-membered ring, Zr_2_AlH_3_. Complex **85** proved to be unstable, and its decomposition at 40 °C over 2–3 h provided red needle-like crystals of compound **85**. For complex **85**, the ^1^H NMR spectrum in d_8_-THF at room temperature exhibited a broad singlet at δ_H_ −2.0 ppm (w_1/2_ ≈ 200 Hz, Al-H-Zr) and a narrow singlet at δ_H_ −7.96 ppm (Zr-H-Zr). The addition of a catalytic amount of CoBr_2_ increased the yield of complex **85** to 25% [103].

**Scheme 46:**
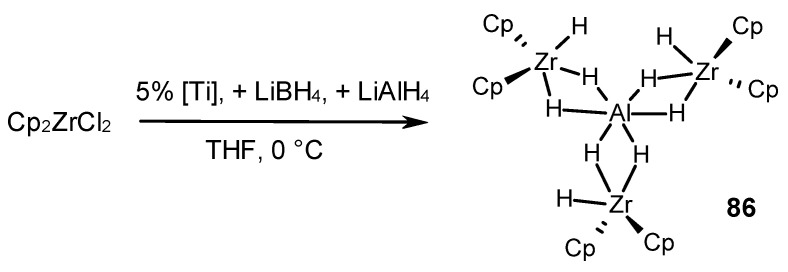
Synthesis of heterometallic complex: [Cp*_2_*ZrH(*µ*-H)*_2_*]*_3_*Al (**86**).

Complex [Cp***_2_***ZrH(*µ*-H)***_2_***]***_3_***Al (**86**), similar to structure **81a**, was obtained by the reaction of Cp_2_ZrCl_2_ with LiBH_4_ and LiAlH_4_ in the presence of 5 mol% Cp_2_TiCl_2_ at 0 °C in THF (Scheme 46) [102]. The structure of complex **86** was determined using the X-ray diffraction method.

**Scheme 47:**
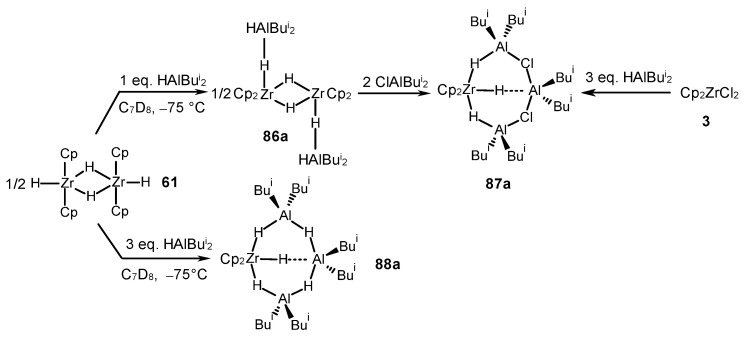
Reactions of Cp_2_ZrCl_2_ (**3**) and [Cp_2_ZrH_2_]_2_ (**61**) with HAlBu^i^_2_ [105].

The reaction of Cp_2_ZrCl_2_ (**3**) with an excess of HAlBu^i^_2_ (in a 1:3 ratio) in d_6_-benzene at 25 °C was accompanied by the formation of tetranuclear trihydride complex **87a**. The complex is characterized by triplet signals at δ_H_ −0.89 ppm (Zr-H, *J* = 7.4 Hz) and doublet signals at δ_H_ −2.06 ppm (Zr-H-Al, *J* = 6.8 Hz) (Scheme 47) [105]. The replacement of zirconocene dichloride with dihydride [Cp_2_ZrH_2_]_2_ (**61**) in the reaction with HAlBu^i^_2_ (at a ratio of [Zr]:[Al] = 1:1) at −75 °C provided initially intermediate **86a**, which, after reacting with ClAlBu^i^_2_, transformed into complex **87a** (Scheme 47). The ^1^H NMR spectrum of compound **86a** exhibited broadened signals of Zr-H hydride atoms at δ_H_ −2.11 and −3.05 ppm. In the case of an excess of the OAC (more than 3 equiv.), structure **88a** was formed at −75 °C to 0 °C. For this compound, the ^1^H NMR spectra displayed signals: a doublet at δ_H_ −2.32 ppm (*J* = 16.5 Hz, Zr-H), a triplet at δ_H_ −1.46 ppm (*J* = 15.5 Hz, Zr-H), and a signal at δ_H_ −3.18 ppm (Al-H-Al) with an integral intensity ratio of 2:1:2. Upon raising the temperature to 25 °C, the proton signals of complex **88a** broadened, indicating its propensity for exchange reactions.

**Scheme 48:**
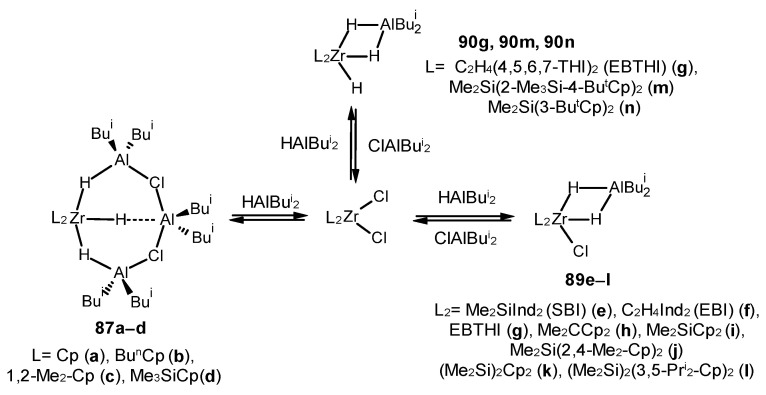
Reactions of various L_2_ZrCl_2_ with HAlBu^i^_2_ [105].

The formation of the L_2_Zr(µ-H)_3_(AlBu^i^_2_)_3_(*µ*-Cl)_2_ (**87b**–**d**) structures is characteristic for L_2_ZrCl_2_ complexes containing unbound cyclopentadienyl ligands (L = Bu^n^-C_5_H_4_ (**b**), 1,2-Me_2_-C_5_H_3_ (**c**), or Me_3_Si-C_5_H_4_ (**d**)) (Scheme 48). The doublet signals of the hydride atoms of Zr-H bonds at δ_H_ −2.09–−1.13 ppm and triplet signals at δ_H_ −1.31–−0.20 ppm were detected in the ^1^H NMR spectra of compounds **87b**–**d**. In this case, the signals of the protons of a Zr-H bond of compounds **87b**–**d** were shifted downfield by 0.42–0.90 ppm compared to **87a** due to a change in the electron density in the substituted cyclopentadienyl ligands [105].

The reactions of the *ansa*-complexes, including (SBI)ZrCl_2_, (EBI)ZrCl_2_, (EBTHI)ZrCl_2_, Me_2_CCp_2_ZrCl_2_, Me_2_SiCp_2_ZrCl_2_, Me_2_Si(2,4-Me_2_-Cp)_2_ZrCl_2_, (Me_2_Si)_2_Cp_2_ZrCl_2_, and (Me_2_Si)_2_(3,5-Pr^i^_2_-Cp)_2_ZrCl_2_, with 2–5 equiv. of HAlBu^i^_2_ in toluene or benzene at room temperature provided hydride complexes **89e**–**l**, whose ^1^H NMR spectra showed the broadened signals of hydride atoms of the [Zr-H]_2_ fragment at δ_H_ −1.75–−0.80 ppm (Scheme 48) [105].

The bulky *tert*-butyl groups in the *ansa*-complexes, *rac*-Me_2_Si(2-Me_3_Si-4-Me_3_C-Cp)_2_ZrCl_2_ and *meso*-Me_2_Si(3-Me_3_C-Cp)_2_ZrCl_2_, led to the formation of trihydride intermediates **90m** and **90n**. The signals of three nonequivalent protons were observed in the ^1^H NMR spectrum of compound **90m** at δ_H_ −1.56, −0.60 (d, *J* = 8.2 Hz, Zr-H-Al), and 2.68 ppm (dd, *J* = 5.5 and 9.4 Hz, Zr-H). The signals at δ_H_ −2.17 (d, J = 5 Hz, Zr-H-Al), −0.21 (d, *J* = 10.2 Hz, Zr-H-Al), and 3.31 ppm (dd, *J* = 5.1 and 9.9 Hz, Zr-H) were detected in the ^1^H NMR spectrum of compound **90n** (Scheme 48) [105].

**Scheme 49:**
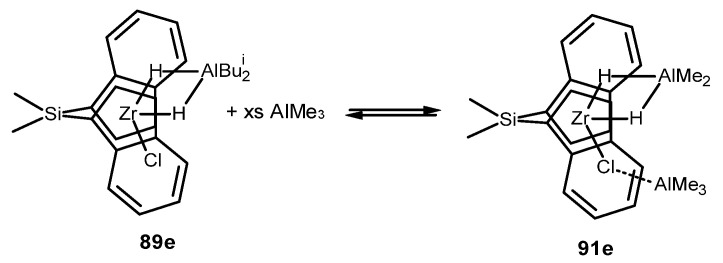
Reaction of complex **89e** with AlMe_3_ [105].

The interaction of (SBI)ZrCl(µ-H)_2_AlBu^i^_2_ (**89e**) with an excess of AlMe_3_ (at a [Zr]:[AlMe_3_] ratio of 1:128) provided complex (SBI)ZrCl(*µ*-H)_2_AlMe_2_ (**91e**) (Scheme 49) [105]. Upon the addition of AlMe_3_, the signal of a Zr-H bond proton in the ^1^H NMR spectrum shifted from δ_H_ 1.22 to 1.65 ppm but did not completely disappear. Therefore, it was concluded that the resulting compound (**91e**) is presumably an adduct containing AlMe_3_ coordinated to the terminal Cl atom in (SBI)ZrCl(µ-H)_2_AlMe_2_, rather than the desired product of exchanging the Me group for a chlorine atom.

**Scheme 50:**
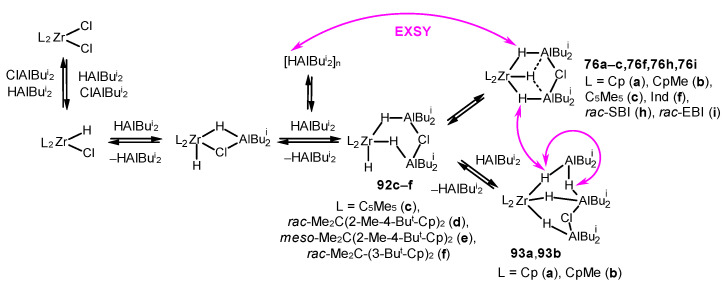
Zr,Al–hydride intermediates generated in the reaction of L_2_ZrCl_2_ with HAlBu^i^_2_ (the pink arrows show the hydride atom exchange observed by NMR) [93,106].

Further, it was established that in the reaction of L_2_ZrCl_2_ with HAlBu^i^_2_ (at a Zr:Al ratio of 1:3), the L*_2_*Zr(μ-H)_3_(AlBu^i^_2_)_2_(μ-Cl) (**76a**–**c**, **76f**, **76h**, and **76i**) and Cp_2_Zr(μ-H)_3_(AlBu^i^_2_)_3_(μ-Cl)(μ-H) (**93a** and **93b**) complexes are predominantly formed (Scheme 50) [93,106]. Using EXSY spectroscopy, the exchange between the hydride atoms of complexes **76** and **93** and oligomers [HAlBu^i^_2_]_n_ was demonstrated. It was assumed that the exchange can proceed through the dissociation of Zr,Al–hydride complexes with the elimination of a HAlBu^i^_2_ monomer.

The dependence of the structure of the intermediates on the nature of the ligand in the initial zirconocene was also demonstrated (Scheme 50) [106]. Metallocenes with bulky η*^5^*-ligands provided structures **92c**–**f** with a terminal Zr-H bond. ^1^H NMR spectra of complexes with sterically hindered ligands, for example, **92c**, recorded at 220 K, exhibited signals of bridging hydrogen atoms at δ_H_ −1.27 ppm and δ_H_ −0.66 ppm (d, *J* = 9.6 Hz) (Zr-H-Al), and the signal of the terminal hydride atom at δ_H_ 4.38 ppm (dd, *J* = 9.6 Hz, 4.0 Hz). Moreover, the EXSY spectra showed cross-peaks between the [HAlBu^i^_2_]_n_ hydride signals and a downfield broadened signal at 6.85 ppm, which was attributed to the hydride atoms of the free (Cme_5_)_2_ZrHCl. A similar pattern in the NMR spectra was observed for sterically hindered complexes **92d**–**f**: bridging hydride atoms of Zr-H-Al fragments resonated in the upfield region at δ_H_ −1.07–−0.33 ppm, while the signals of a terminal Zr-H bond were shifted to the downfield δ_H_ 2.05–3.51 ppm. The systems based on zirconocene dichlorides with sterically hindered ligands, which provided intermediates **92c**, **92d**, and **92f** with an open Zr-H bond, appeared to be the most active in alkene hydroalumination reaction.

**Scheme 51:**
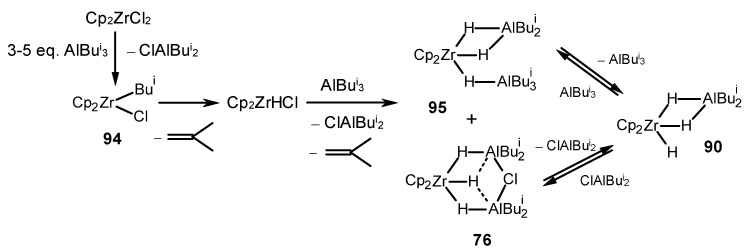
Zr,Al–hydride intermediates generated in the reaction of Cp_2_ZrCl_2_ with AlBu^i^_3_ [106].

In the reaction of Cp_2_ZrCl_2_ with AlBu^i^_3_ (1:5), alkyl chloride complex **94** was detected, which then transformed into complexes **95** and **76**, undergoing intermolecular exchange via intermediate **90** (Scheme 51) [106]. The structure of complex **95** was identified based on the observation of three upfield doublets of magnetically non-equivalent hydride atoms of the bridging Zr-H-Al bonds in a 1:1:1 ratio, at δ_H_ −1.15, −1.83, and −2.48 ppm in the ^1^H NMR spectrum recorded at low temperature (230 K). It is noted that the probable reason for the high reactivity of the Cp_2_ZrCl_2_-AlBu^i^_3_ catalytic system towards alkenes is the absence of fast exchange between hydride clusters, leading to an increase in the lifetime of intermediates with a free Zr-H bond and an absence of opportunity for the formation of larger clusters like complexes **87** and **93**.

Indenyl hydride complexes **76f**, **76h**, and **76i** obtained by the reaction of the corresponding zirconocenes with an excess of HAlBu^i^_2_ also contained a [L_2_ZrH_3_] moiety (Scheme 50). The hydride atoms were in fast exchange with [HAlBu^i^_2_]_n_ oligomers; therefore, the signals of hydride atoms in the ^1^H NMR spectra were significantly broadened at room temperature. As the temperature decreased below 280 K, the exchange slowed down, and the multiplet signals of Zr-H-Al hydrides in the ranges of δ_H_ −1.55–−1.00 ppm and δ_H_ 0.62–1.06 ppm were detected in the ^1^H NMR spectra of compounds **76f**, **76h**, and **76i** (Scheme 50) [106]. 

The studies on the catalytic activity of L_2_ZrCl_2_-XAlBu^i^_2_ systems (L = Cp, CpMe, Ind, C_5_Me_5_; L_2_ = *rac*-Me_2_C(2-Me-4-Bu^t^-Cp)_2_; *meso*-Me_2_C(2-Me-4-Bu^t^-Cp)_2_; *rac*-Me_2_C(3-Bu^t^-Cp)_2_; *rac*-Me_2_C(Ind)_2_; *rac*-Me_2_Si(Ind)_2_ (SBI); *rac*-C_2_H_4_(Ind)_2_ (EBI)); and X = H, Cl, and Bu^i^) in the alkene hydroalumination showed that the maximum effect is achieved when complexes with more bulky cyclopentadienyl ligands are used in combination with HAlBu^i^_2_. The catalysts with less bulky ligands are the most active in the reaction of alkenes with AlBu^i^_3_ or ClAlBu^i^_2_. Indenyl zirconium complexes provide a significant decrease in the yield of hydroalumination products, regardless of the structure of OACs. This dependence of the activity of a catalytic system on the nature of OAC and the structure of a ligand in zirconocene is due to the structural and dynamic features of bimetallic hydride intermediates formed in these systems [106].

**Scheme 52:**
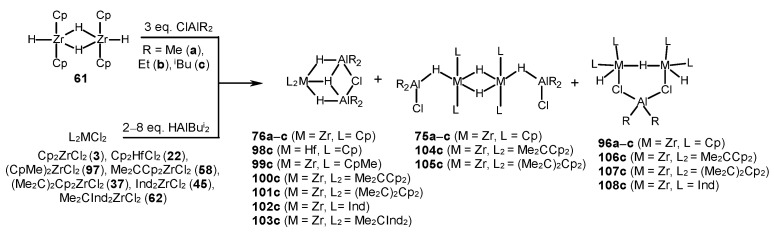
Zr,Al–hydride intermediates observed in the [Cp_2_ZrH_2_]_2_-ClAlR_2_ and L_2_ZrCl_2_-HAlBu^i^_2_ systems [71,74,75,93].

Complexes **96a**–**c**, together with the intermediates **75a**–**c** and **76a**–**c** (Scheme 52), were observed in the [Cp_2_ZrH_2_]_2_-ClAlR_2_ systems (R = Me (**a**), Et (**b**), and Bu^i^ (**c**)) [71,74,93]. The spectral pattern for complexes **96a**–**c** differed significantly from those of **75a**–**c** and **76a**–**c**. The ^1^H NMR spectra of complexes **96a**–**c** exhibited distinct triplet upfield signals at δ_H_ −6.64–−6.35 ppm (*J* = 17.0–17.6 Hz) assigned to a hydride atom of the Zr-H-Zr bond. This signal in the COSY HH spectrum correlated with a doublet at −1.39–−1.18 ppm (*J* = 17.0–17.6 Hz), with a ratio of integral intensities, 1(Zr-H-Zr):2(Zr-H-Al):20(Cp), which indicated the presence of the [(L_2_Zr)_2_H_3_] moiety in the molecule. Complex **96c**, along with **75c** and **76c**, was also detected in minor amounts in the reaction of Cp_2_ZrCl_2_ with HAlBu^i^_2_ at a low OAC content (Scheme 52).

For the Cp_2_HfCl_2_ (**22**), (CpMe)_2_ZrCl_2_ (**97**), Me_2_CCp_2_ZrCl_2_ (**58**), (Me_2_C)_2_Cp_2_ZrCl_2_ (**37**), Ind_2_ZrCl_2_ (**45**), and Me_2_CInd_2_ZrCl_2_ (**62**) complexes, the reaction with HAlBu^i^_2_ also resulted in the formation of structures **98**–**105c** at a [Al]/[Zr] ratio of 3–8 [74,75]. Intermediates **106**–**108c** were observed at low AOC contents in the system ([Al]/[Zr] = 2–3).

**Scheme 53:**
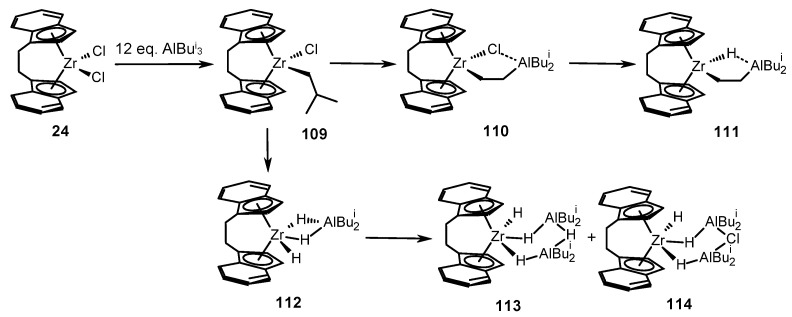
Reaction of *ansa*-zirconocene (EBI)ZrCl_2_ with AlBu^i^_3_ [107].

Recently, it has been demonstrated that the reaction of *ansa*-zirconocene (EBI)ZrCl_2_ with an excess of AlBu^i^_3_ (in a 1:12 ratio) in d_6_-benzene at 25 °C for 10 min provided a mixture of complexes: (EBI)ZrBu^i^Cl (**109**) (95%) and (EBI)Zr(µ-Cl)(µ-CH_2_CH_2_)AlBu^i^_2_ (**110**) (5%) (Scheme 53) [107]. Complexes **109** and **110** transformed into hydride intermediates (EBI)Zr(μ-H)(μ-CH_2_CH_2_)AlBu^i^_2_ (**111**) and (EBI)ZrH(μ-H)_2_AlBu^i^_2_ (**112**) after 3 h of the reaction. In the ^1^H NMR spectrum of complex **111**, the broadened singlet signal of a proton of a Zr-H-Al fragment at δ_H_ −2.62 ppm, multiplet signals of protons of the Zr(*µ*-CH_2_CH_2_)AlBu^i^_2_ bridge at δ_H_ −2.12, −1.58, 0.14, and 1.17 ppm correlated with the signals in the ^1^H-^13^C HSQC spectra at δ_C_ 4.7 ppm (Al-CH_2_) and 53.4 ppm (Zr-CH_2_), were observed (Scheme 46). Complex **112** was characterized by the signals of hydrides at δ_H_ −1.77 (d, *J*_HH_ = 6.3 Hz) and −1.44 ppm that correlated with a proton signal at δ_H_ −0.22 ppm in COSY HH spectra. The hydride complexes (EBI)ZrH(μ-H)_2_[μ-H(AlBu^i^_2_)_2_] (**113**) and (EBI)ZrH(μ-H)_2_[μ-Cl(AlBu^i^_2_)_2_] (**114**) were detected after 16 h of the experiment. Complexes **111**–**114** were the major products even after 40 h of reaction (Scheme 53).

### 3.3. Influence of Al- and B-Containing Activators on Structure and Reactivity of Metallocene Hydrides

**Scheme 54:**
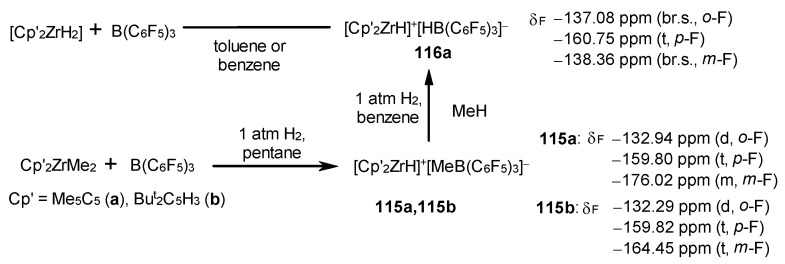
Reactions of Cp′_2_Zr(CH_3_)_2_ and Cp′_2_ZrH_2_ with B(C_6_F_5_)_3_ at −78 °C in the presence of H_2_ [27,108].

Aluminum- and boron-containing activators have significant effects on the structure and reactivity of intermediates formed in metallocene systems. For example, the hydride complexes [Cp′_2_ZrH]^+^[MeB(C_6_F_5_)_3_]^−^ (**115a**,**b**) and [Cp′_2_ZrH]^+^[HB(C_6_F_5_)_3_]^−^ (**116a**) were observed in the reaction of Cp′_2_Zr(CH_3_)_2_ or Cp’_2_ZrH_2_ (Cp′ = η*^5^*-Me_5_C_5_ (**a**) and η*^5^*-Bu^t^_2_C_5_H_3_) (**b**)) with B(C_6_F_5_)_3_ at −78 °C in the presence of H_2_ (1 atm) (Scheme 54) [27,108]. In the ^1^H NMR spectra of complexes **115a** and **116a**, the hydride atoms of a Zr-H bond resonated at δ_H_ 7.70 and 8.18 ppm, respectively. The broadened singlet signals at δ_H_ 0.10 ppm (B-CH_3_, complex **115a**) and δ_H_ 3.98 ppm (B-H, complex **116a**) were also detected in the ^1^H NMR spectrum. Compounds **115a** and **116a** turned out to be active homogeneous catalysts for the polymerization of ethylene (3.2·10^6^ g_PE_ mol_Zr_^−1^ h^−1^ atm^−1^, M_W_ = 4.34·10^5^) and propylene (3.2·10^5^ g_PP_ mol_Zr_^−1^ h^−1^, M_W_ = 3900).

**Scheme 55:**
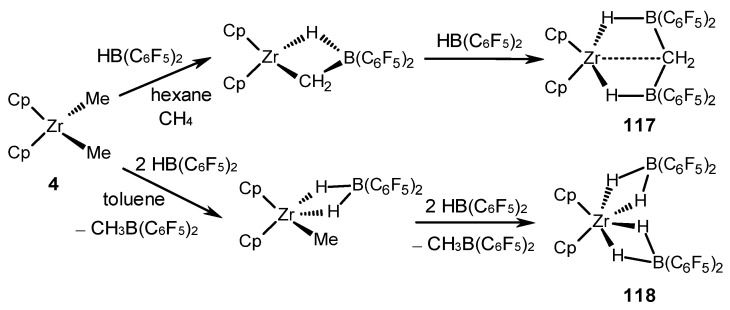
The reaction of Cp_2_ZrMe_2_ with HB(C_6_F_5_)_2_Zr to give borohydride complexes **117** and **118**.

Zr borohydride complexes **117** and **118** were synthesized by the reaction of alkyl zirconocenes with HB(C_6_F_5_)_2_ (Scheme 55) [109,110]. The formation of complex **117** in the reaction of Cp_2_ZrMe_2_ with HB(C_6_F_5_)_2_ was monitored by NMR spectroscopy through the evolution of CH_4_ (δ_H_ 0.16 ppm) and the appearance of a brick-red precipitate at the bottom of a tube (Scheme 55). In the ^1^H NMR spectra of complex **117** in hexane, signals of Cp-ring protons were observed at δ_H_ 5.23 ppm, signals of CH_2_ fragment hydrogen atoms were observed at δ_H_ 2.29 ppm, and broadened signals of Zr-H-B bridging hydrides were observed at δ_H_ −2.05 ppm in a ratio of 10:2:2. In the ^13^C NMR spectrum of compound **117**, signals of Cp rings and the CH_2_ group were detected at δ_C_ 111.11 ppm and δ_C_ 0.5 ppm (^1^*J*_C-H_ = 120 Hz), respectively. The fluorine atoms of a B(C_6_F_5_)_2_ group resonated at δ_F_ −132.4, −157.2, and −163.4 ppm in the ^19^F NMR spectra. The ^11^B NMR spectrum exhibited a signal at δ_B_ 0.00 ppm (^1^*J*_H-B_ = 135 Hz), which is typical of a four-coordinated boron atom. The structure of complex **117** was also confirmed by X-ray diffraction. The replacement of an aliphatic solvent with toluene and an increase in the amount of HB(C_6_F_5_)_2_ to two equivalents led to the formation of complex **118**. In the ^1^H NMR spectra, the signals of Cp rings and a Zr-H-B hydride atom were shifted to the upfield to δ_H_ 5.42 and 0.38 ppm, respectively, compared to structure **117**. A triplet signal also appeared at δ_B_ −12.9 ppm (^1^*J*_H-B_ = 64 Hz) in the ^11^B NMR spectra. The ^19^F NMR spectrum of compound **118** (δ_F_ −133.0, −156.8, and −163.4 ppm) remained almost unchanged compared to that of complex **117**. It turned out that complex **118** was inactive in ethylene polymerization.

**Scheme 56:**
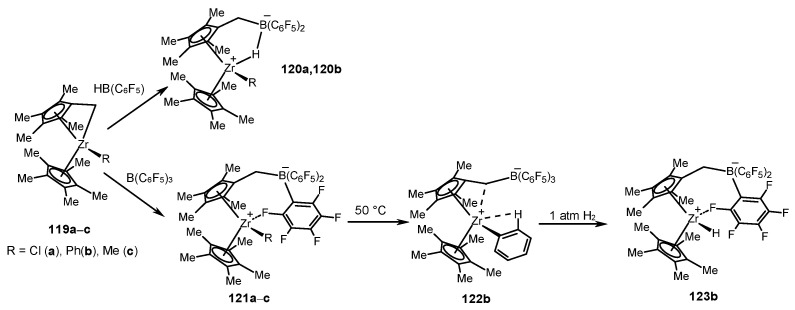
Reaction of Cp*(η^5^-η^1^-C_5_Me_4_CH_2_)ZrR compounds with highly electrophilic boranes HB(C_6_F_5_)_2_ and B(C_6_F_5_)_3_.

The Cp*(η^5^-η^1^-C_5_Me_4_CH_2_)ZrR **119a**–**c** compounds (R = Cl, CH_3_, and C_6_H_5_) in reaction with highly electrophilic boranes, HB(C_6_F_5_)_2_ and B(C_6_F_5_)_3_, provided the following hydride cationic complexes: Cp*(η^5^-η^1^-C_5_Me_4_CH_2_B(C_6_F_5_)_2_(*µ*-H)ZrR (**120a**: R = Cl with 74% yield; **120b**: R = C_6_H_5_, 62% yield) and Cp*[η*^5^*-C_5_Me_4_CH_2_B(C_6_F_5_)_3_]ZrH (**123b**, 77% yield) (Scheme 56) [111]. The ^1^H NMR spectrum of **120a** showed the doublet and the doublet of doublet signals of a CH_2_ group at δ_H_ 3.11 and 2.88 ppm, respectively, as well as broadened signals of a Zr-H-B fragment at δ_H_ 0.5 ppm. For hydride complex **123b**, obtained from Cp*(η^5^-η^1^-C_5_Me_4_CH_2_)ZrPh (**119b**) and B(C_6_F_5_)_3_ through a series of stages, as depicted in Scheme 56, the presence of hydrogen atom signals of CH_2_B moiety in the ^1^H NMR spectra at δ_H_ 2.66 and 3.13 ppm is characteristic. Upon increasing the temperature to 50 °C, the Cp*(η^5^-η^1^-C_5_Me_4_CH_2_)ZrPh compound (**119b**) in the NMR tube converted to product **122b**, which then transformed into complex **123b** after hydrogen bubbling. Compounds **120a**, **121a**, **122b**, and **123b** proved to be active catalysts in the ethylene polymerization reaction.

**Scheme 57:**
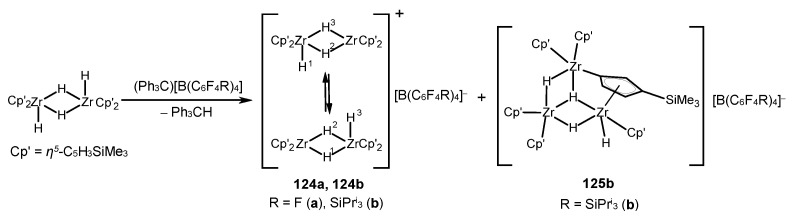
Zirconium hydride intermediates obtained in the reaction of [Cp′_2_ZrH_2_]_2_ with (Ph_3_C)[B(C_6_F_4_R)_4_].

Binuclear hydride complexes [Cp′_4_Zr_2_H_3_][B(C_6_F_4_R)_4_] **124a** and **124b** (R = F (**a**) and SiPr^i^_3_ (**b**)) were obtained by the reaction of [Cp′_2_ZrH_2_]_2_ with a solution of (Ph_3_C)[B(C_6_F_4_R)_4_] in d_8_-toluene (Scheme 57) [112]. Two signals of bridging hydrides H^1^ and H^2^ at δ_H_ −2.02 and −2.66 ppm and terminal H^3^ protons at δ_H_ 4.55 ppm were detected in the ^1^H NMR spectrum of complex **124a** at −78 °C. All three signals coalesced at −30 °C due to a fast hydride exchange. The [Cp′_2_ZrH_2_]_2_-(Ph_3_C)[B(C_6_F_4_R)_4_] system turned out to be much more active in the homopolymerization of isobutene and the isobutene–isoprene copolymerization compared to the system based on Cp′_2_ZrMe_2_. Complex **124b**, also obtained in the reaction of [Cp′_2_ZrH_2_]_2_ with (Ph_3_C)[B(C_6_F_4_SiPr^i^_3_)_4_] in a [Zr]:[B] ratio of 1:1, transformed into pale yellow-green crystals of compound **125b** over several days at 5 °C. The structure of the complex was confirmed by X-ray crystallography [112].

Using NMR spectroscopy, it was demonstrated that in the reaction of Ph_2_C(CpFlu)ZrCl_2_ with AlBu^i^_3_ in the presence of (PhNMe_2_H)[B(C_6_F_5_)_4_] with a [Zr]:[Al]:[B] reactant ratio of 1:(10–100):1 in d_6_-benzene at 60 °C, the isobutyl derivative, [Ph_2_C(CpFlu)ZrBu^i^·AlBu^i^_3_]^+^, transforms into the allyl hydrido complex, [Ph_2_C(CpFlu)Zr(μ-H)(μ-C_4_H_7_)AlBu^i^_2_][B(C_6_F_5_)_4_] (**68**) (Scheme 34) [67]. The diastereotopic protons of a Zr–CH_2_ bond resonated at δ_H_ 2.87 and −1.66 ppm, and the hydrogen atoms of an Al-CH_2_ fragment resonated at δ_H_ 2.28 ppm and around 1 ppm in the ^1^H NMR spectrum. The signals correlated with resonance lines at δ_C_ 90.5 ppm (ZrCH_2_, ^1^J_C-H_ = 157.5 Hz) and 47.7 ppm (AlCH_2_, ^1^J_C-H_ = 129.4 Hz) in the ^13^C NMR spectrum; this indicates the non-symmetric bonding of the allyl moiety. The signal at δ_C_ 163.4 ppm was attributed to the quarternary methallyl C atom. The hydride atom of a Zr-H-Al bridge resonated at δ_H_ −2.78 ppm in the ^1^H NMR spectrum of compound **68**. In the ^19^F spectrum of the compound, signals for the [B(C_6_F_5_)_4_]^−^ anion were present as a broad singlet at δ_F_ −131.8 ppm (*o*-F), a triplet at δ_F_ −162.4 ppm (*J* = 20.4 Hz, *p*-F), and a multiplet at δ_F_ −166.2 ppm (*m*-F), which designated the lack of coordination of the anion with the cation.

**Scheme 58:**
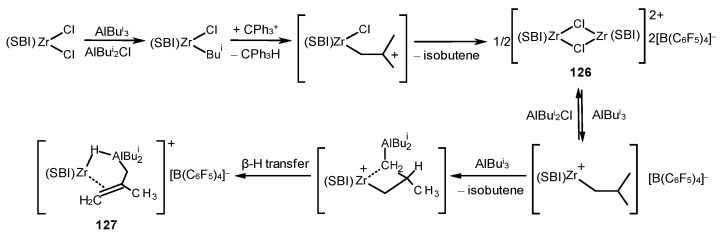
Reaction of (SBI)ZrX_2_ (X = Cl and Me) with AlBu^i^_3_ in the presence of (Ph_3_C)[B(C_6_F_5_)_4_] [66].

The [(SBI)Zr(*µ*-Cl)_2_Zr(SBI)][B(C_6_F_5_)_4_]_2_ (**126**) and [(SBI)Zr(*µ*-H)(*µ*-C_4_H_7_)AlBu^i^_2_][B(C_6_F_5_)_4_] (**127**) complexes were identified in the reaction of (SBI)ZrX_2_ (X = Cl and Me) with AlBu^i^_3_ in the presence of (Ph_3_C)[B(C_6_F_5_)_4_] (Scheme 58) [66]. Initially, upon the interaction of (SBI)ZrCl_2_ and AlBu^i^_3_ at [Zr]:[Al] = 1:(5–10) with the addition of 1 eq. of Ph_3_C[B(C_6_F_5_)_4_] over 5–15 min, ionic dimeric structure **126** occurred, which was characterized using X-ray crystallography (Scheme 58). Complex **127** was formed through several stages at a reagent ratio of (SBI)ZrCl_2_ and AlBu^i^_3_ [Zr]:[Al] of 1: ≥20 at room temperature. In the ^1^H NMR spectrum of structure **127**, singlet proton signals were observed at δ_H_ 3.03 (Zr-C*H*H) and −1.73 ppm (Zr-CH*H*), at δ_H_ 2.51 (Al-C*H*H) and 0.22 ppm (Al-CH*H*), as well as at δ_H_ −3.35 ppm (Zr-H-Al). The resonance lines of the C atoms of Zr-CH_2_ and Al–CH_2_ were located at δ_C_ 86.9 and 53.8 ppm, respectively. A chemical shift of the CH_2_ = CMe moiety equal to 166.6 ppm was characteristic of a methallylic structure. It is noted that in the presence of excess AlBu^i^_3_, species **126** serves as precursor for propylene polymerization active sites, whereas species **127** is a thermodynamic sink of the catalytic system.

**Scheme 59:**
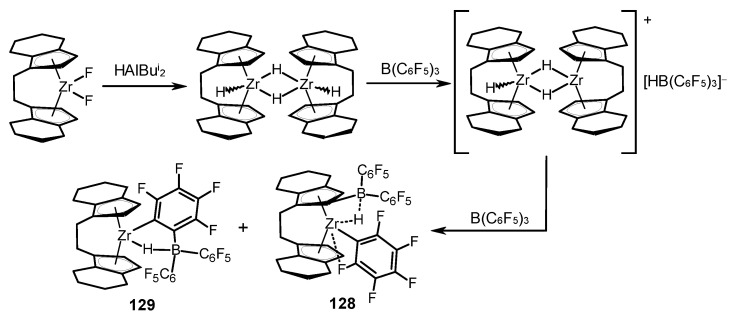
Reaction of *rac*-ethylenebis(4,5,6,7-tetrahydro-1-indenyl) zirconium difluoride with HAlBu^i^_2_ and B(C_6_F_5_)_3_ [113,114].

Zr,B-hydride complexes **128** and **129** were obtained in the reaction of *rac*-ethylenebis(4,5,6,7-tetrahydro-1-indenyl) zirconium difluoride with HAlBu^i^_2_ and B(C_6_F_5_)_3_ (Scheme 59) [113,114]. Compounds **128** and **129** were identified using X-ray crystallography and NMR spectroscopy. It has been demonstrated that a catalytic system based on metallocene fluorides and AlBu^i^_3_ provides hydride-containing Zr complexes, exhibiting excellent activity in the polymerization of ethylene and propylene.

**Scheme 60:**
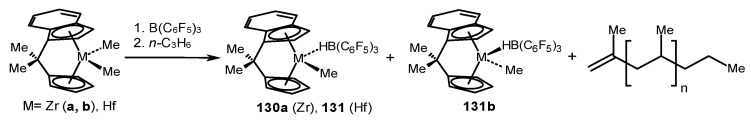
Reaction of dialkyl Me_2_C(Cp)IndMMe_2_ complexes with B(C_6_F_5_)_3_ [115].

The Me_2_C(Cp)IndMMe(µ-H)B(C_6_F_5_)_3_ (M = Zr and Hf) **130a**, **130b**, **131** hydride intermediates were observed in the reaction of Me_2_C(Cp)IndMMe_2_ dialkyl complexes with B(C_6_F_5_)_3_ (Scheme 60) [115]. Two isomeric structures, **130a** and **130b**, along with an oligomerization product, were detected in the case of a Zr–borohydride complex, Me_2_C(Cp)IndZrMe(*µ*-H)B(C_6_F_5_)_3_, obtained at a [Zr]:[B] reagent ratio of 1:1.2 at 25 °C in d_8_-toluene with the addition of 10 eq. of propylene. In the ^1^H NMR spectrum of major isomer **130a**, signals corresponding to the protons of the Zr-Me bond were observed at δ_H_ −1.10 ppm (septet, ^3^*J*_H-F_ = 2.2 Hz). The following ^19^F NMR signals were typical for isomer **130a**: a broadened doublet at δ_F_ −131.0 (*J* = 18.3 Hz, *o*-F), a triplet at δ_F_ −155.0 (*J* = 21.4 Hz, *p*-F), and a multiplet at δ_F_ −162.0 ppm (*m*-F). The ^1^H NMR spectrum of minor isomer **130b** exhibited the signals of protons of a Zr-Me bond at δ_H_ 0.27 ppm. The ^19^F NMR spectrum of compound **130b** showed minor differences compared to **130a**: a broadened doublet at δ_F_ −132.2 (*J* = 18.3 Hz, *o*-F), a triplet at δ_F_ −156.5 (*J* = 21.4 Hz, *p*-F), and a multiplet at δ_F_ −162.8 ppm (*m*-F).

The *ansa*-hafnocene hydride complex **131** was characterized by X-ray diffraction and NMR spectroscopy as well (Scheme 60) [115]. In the ^1^H NMR spectrum, a signal corresponding to the Hf-Me bond was observed at δ_H_ −1.10 ppm (septet, ^3^*J*_H-F_ = 2.2 Hz), and signals of the Hf-H-B fragment were found at δ_H_ 0.44 ppm, identified through correlation in the ^1^H−^11^B spectra. The following signals of the HB(C_6_F_5_)_3_ group of compound **131** were detected in the ^19^F NMR spectra: a doublet at δ_F_ −130.7 (*J* = 24.4 Hz, *o*-F), a triplet at δ_F_ −155.0 (*J* = 21.4 Hz, *p*-F), and a multiplet at δ_F_ −162.2 ppm (*m*-F). Intermediates **130a**, **130b**, and **131** were found to be relatively inert towards propene and were in an inactive “dormant” state. 

**Scheme 61:**
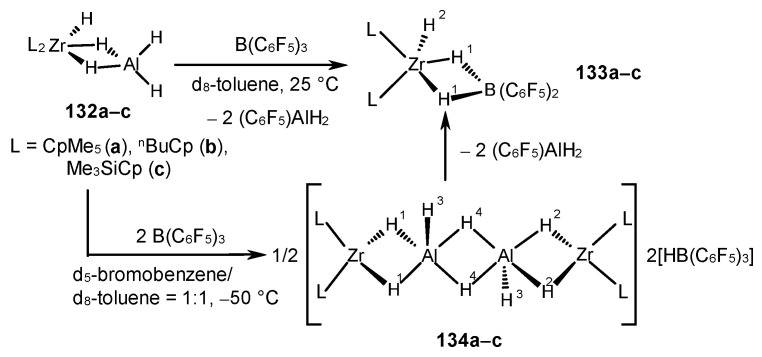
Reaction of Zr,Al–hydride complexes L_2_ZrH_3_AlH_2_ (**132a**–**c**) with B(C_6_F_5_)_3_ [116].

Zr,Al–hydride L_2_ZrH_3_AlH_2_ complexes (**132a**–**c**) (L = CpMe_5_ (**a**), Bu^n^Cp (**b**), and Me_3_SiCp (**c**)) formed the metallocene di- or polynuclear ion pairs with HB(C_6_F_5_)_3_^−^ (**134a**–**c**) upon the activation with B(C_6_F_5_)_3_ at −50 °C in a 1:1 mixture of d_5_-bromobenzene and d_8_-toluene (Scheme 61) [116]. The dinuclear structure of the ion pair **134a** was confirmed by the presence of two distinct signals of a C_5_Me_5_ ligand in a 1:1 ratio and broadened doublets of bridging hydrides of Zr-H^1,2^-Al bonds at δ_H_ −2.94 and −2.13 ppm, as well as terminal protons of the Al-H^3^ bonds at δ_H_ 4.16 ppm in the ^1^H NMR spectra. The broadened signals of the Al-H^4^-Al moiety were also observed at δ_H_ 0.4 ppm. The ^19^F NMR spectrum showed the characteristic signals of an HB(C_6_F_5_)_3_^−^ anion at δ_F_ −133.0, −163.2, and −166.3 ppm (**134a**). Complex **134a** transformed into L_2_ZrH(*µ*-H)_2_B(C_6_F_5_)_2_ (**133a**) and (C_6_F_5_)AlH_2_ as a result of thermal decomposition (Scheme 61). The same products were generated in the reaction of **132a** with B(C_6_F_5_)_3_ in toluene. A singlet signal at δ_H_ 6.64 ppm and quartet signals of bridge hydrides at δ_H_ −0.73 ppm (*J*_B-H_ = 75 Hz, Zr-H^1^) were observed in the ^1^H NMR spectrum of structure **133a**. It has been shown that the two bridge hydride atoms are in rapid exchange between the central and side positions, but neither of them exchanges with a terminal hydride of a Zr-H^2^ bond. A broadened doublet at δ_F_ −130.3 (*o*-F), a triplet at δ_F_ −157.6 (*p*-F), and a multiplet at δ_F_ −163.6 ppm (*m*-F), which are characteristic of a tetrahedral fragment H_2_B(C_6_F_5_)_2_^–^ coordinated with Zr, were detected in the ^19^F NMR spectrum. The obtained hydride complex **133a** exhibited moderate activity in the ethylene polymerization reaction (activity—4 · 10^3^ g_PE_ mol^−1^ h^−1^ at 25 °C and 2.7 atm). However, the catalyst formed as a result of complex **132a** activation with B(C_6_F_5_)_3_ proved to be 1000 times more active than **133a**. Complex **132c** also resulted in the generation of a more active catalyst at elevated temperatures upon activation with B(C_6_F_5_)_3_. The authors explained this by the higher thermal stability of the particles associated with the bridging anion, HB(C_6_F_5_)_3_^−^ [116].

**Scheme 62:**
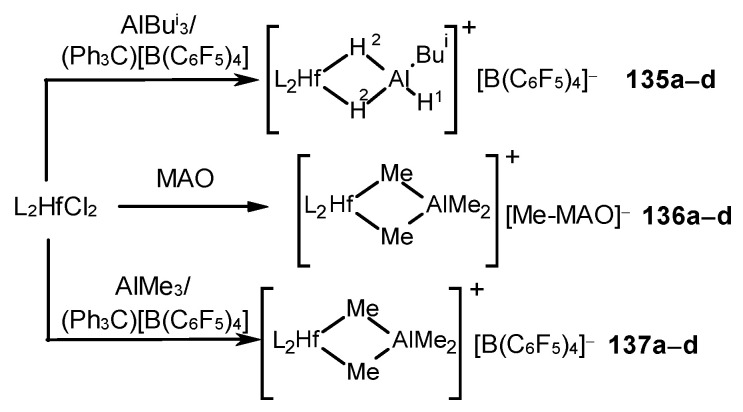
Cationic intermediates observed in the reaction of hafnocenes with AlBu^i^_3_/(Ph_3_C)[B(C_6_F_5_)_4_] [117].

The interaction of L’HfCl_2_ hafnocenes (L = (SBI) (**a**), Me_2_C(C_5_H_4_)(Flu) (**b**), Ph_2_C(C_5_H_4_)(Flu) (**c**), and C_2_H_4_(Flu)(5,6-C_3_H_6_-2-MeInd) (**d**)) with AlBu^i^_3_/(Ph_3_C)[B(C_6_F_5_)_4_] provided cationic intermediates, [LHf(μ-H)_2_AlBu^i^_2_]^+^ or [LHf(μ-H)_2_Al(H)Bu^i^]^+^ (**135a**–**d**), which showed greater activity in the alkene polymerization than the heterobinuclear methyl-bridged intermediates, [LHf(μ-Me)_2_Al(μ-Me)_2_][MeMAO] (**136a**–**d**) and [LHf(μ-Me)_2_Al(μ-Me)_2_][B(C_6_F_5_)_4_] (**137a**–**d**) (Scheme 62) [117]. Complex (SBI)HfCl_2_, in the reaction with AlBu^i^_3_ and (Ph_3_C)[B(C_6_F_5_)_4_] at a [Hf]:[Al]:[B] ratio of 1:(10–50):1.1, gave rise to the viscous product **135a**, which settled at the bottom of the NMR tube. The ^1^H NMR spectrum of compound **135a** showed two signals of hydride atoms at δ_H_ −1.13 ppm (d, ^2^*J*_HH_ = 5 Hz, H^2^) and δ_H_ 1.40 ppm (t, ^2^*J*_HH_ = 5 Hz, H^1^), which were correlated in the COSY HH spectra. Complexes **135b** and **135c** were unstable at 2–5 °C. Hydride complex **135d**, obtained at a [Hf]:[Al]:[B] reagent ratio of 1:(40–100):1, was characterized by a signal of an H^1^ proton at δ_H_ −2.11 ppm (d, *J*_HH_ = 6 Hz) and a signal of hydrogen atoms H^2^ at δ_H_ −4.00 ppm (dd, *J*_HH_ = 10 Hz and 3.7 Hz). The ^19^F NMR spectrum of the complex exhibited the signals of B(C_6_F_5_)_4_^–^ groups at δ_F_ −132.5, −163.0, and −166.5 ppm [117].

**Scheme 63:**
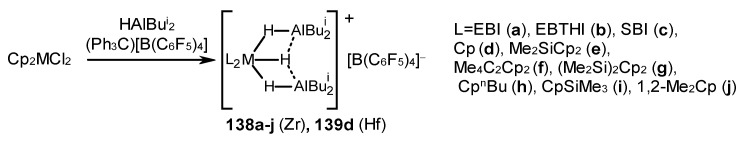
Bimetallic Zr,Al-trihydride cations obtained in L_2_MCl_2_-HAlBu^i^_2_-(Ph_3_C)[B(C_6_F_5_)_4_] catalytic systems [118].

The bimetallic Zr,Al-trihydride cations [L_2_M(*μ*-H)_3_(Al(Bu^i^_2_)_2_]^+^ (**138a**–**j** and **139d**) were obtained in L_2_MCl_2_-HAlBu^i^_2_-(Ph_3_C)[B(C_6_F_5_)_4_] catalytic systems (L = EBI, EBTHI, SBI, Cp, Me_2_SiCp_2_, Me_4_C_2_Cp_2_, (Me_2_Si)_2_Cp_2_, CpBu^n^, CpSiMe_3_, and 1,2-Me_2_Cp; M = Zr and Hf) [118]. In the ^1^H NMR spectra of compounds **138a**–**j**, doublet and triplet signals of three hydride atoms of a ZrH_3_ moiety in the ratio of intensities of 2:1 were observed in the upfield region (Scheme 63). The structures of complexes **138c** and **138g** were confirmed by X-ray diffraction.

**Scheme 64:**
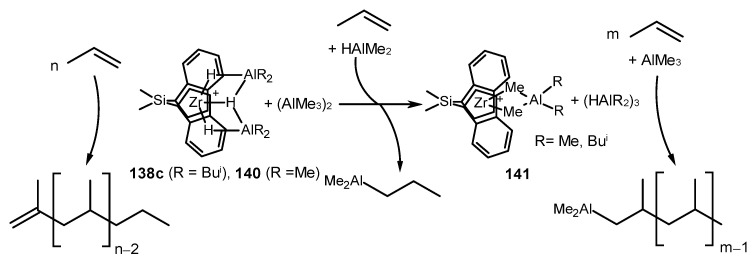
Propene transformations under the actions of complexes **138c**, **140**, and **141** [119].

Complex [(SBI)Zr(μ-H)_3_(AlBu^i^_2_)_2_]^+^ (**138c**) generated in the (SBI)ZrCl_2_–HAlBu^i^_2_-(Ph_3_C)[B(C_6_F_5_)_4_] system at a [Zr]:[Al]:[B]:[propylene] ratio of 1:20:1:20 in d_8_-toluene at −30°C has been shown to polymerize propylene, yielding an isotactic product with 97mmmm% and PDI = 1.90 (Scheme 64) [119]. Polypropylene was also obtained in the presence of a [(SBI)Zr-(μ-Me)_2_AlMe_2_]^+^ cation (**141**) formed in the reaction of complex **140** with (AlMe_3_)_2_. The polymer contained terminal isopropyl groups originated from the chain termination through its transfer to aluminum. After the complete consumption of aluminum hydride, the [(SBI)Zr(μ-Me)_2_AlR_2_]^+^ complex with a dimethyl bridge (**141**) became the sole intermediate in these reaction systems. In the reaction of (SBI)ZrCl_2_ with 20 eq. of HAlMe_2_ and 1 eq. of (Ph_3_C)[B(C_6_F_5_)_4_] in d_8_-toluene, the [(SBI)Zr(μ-H)_3_(AlMe_2_)_2_]^+^ intermediate (**140**) was identified. The cation, [(SBI)Zr(μ-H)_3_(AlBu^i^_2_)_2_]^+^ (**138c**), formed under the action of HAlBu^i^_2_ catalyzed the polymerization of propylene, and its analog, [(SBI)Zr(μ-H)_3_(AlMe_2_)_2_]^+^ (**140**), formed in the presence of HAlMe_2_, showed the activity in propene hydroalumination, transforming during this reaction into the [(SBI)Zr(μ-Me)_2_AlR_2_]^+^ cation (**141**), which also catalyzed the polymerization of propene.

**Scheme 65:**
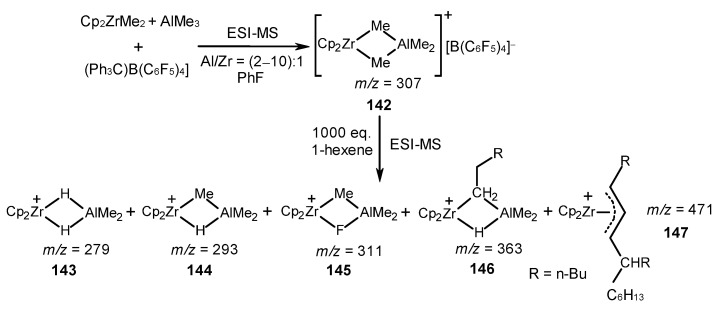
Study of the Cp_2_ZrMe_2_-AlMe_3_-(Ph_3_C)[B(C_6_F_5_)_4_] system using the ESI-MS method [120].

The study of the Cp_2_ZrMe_2_-AlMe_3_-(Ph_3_C)[B(C_6_F_5_)_4_] system (fluorobenzene as a solvent) with the ESI-MS method showed that the main product was the [Cp_2_Zr(μ-Me)_2_AlMe_2_]^+^[B(C_6_F_5_)_4_]^−^ complex (**142**) (Scheme 65) [120]. When 1-hexene is added to the system, complexes **145** and **146**, and the allylic structures [Cp_2_Zr(η*^3^*-C_6_H_10_)(C_6_H_12_)_n_H]^+^ (**147a**,**b**) are formed, and the [Cp_2_Zr(*µ*-H)_2_AlMe_2_]^+^ compound (**143**) with a mass (*m*/*z*) of 279, accumulated as an alkene, is consumed. The formation of dimethylalane hydride structures **144** and **146** is a catalyst deactivation process because a monomer is consumed slowly in the presence of these complexes compared to the starting reaction rates.

There are not much data in the literature on the structures of metallocene hydrides obtained as a result of an interaction with MAO in comparison with hydride complexes activated by B-containing compounds.

It was shown, for example, that aluminum hydride complexes (RCp)_2_ZrH_3_AlH_2_ **132b** and **132c** (R = Bu^n^ (**b**), Me_3_Si (**c**), Scheme 61) activated by MAO possess higher activity in an ethylene polymerization reaction (**132b**: 15.8·10^6^ g_oligomer_ mol_cat_^−1^ h^−1^ and **132c**: 58.1·10^6^ g_oligomer_ mol_cat_^−1^ h^−1^) than the corresponding dichloride complexes, Bu^n^Cp_2_ZrCl_2_ (**40**) and (Me_3_SiCp)_2_ZrCl_2_ (**80**) (**40**: 11.8·10^6^ g_oligomer_ mol_cat_^−1^ h^−1^ and **80**: 43.6·10^6^ g_oligomer_ mol_cat_^−1^ h^−1^) [116]. The molecular weight of the polymer decreased significantly when using a SiO_2_-supported or leached catalyst compared to the corresponding soluble catalyst under the same conditions [121,122]. Polyethylene with M_W_ = 149,500 was obtained in the presence of complex **132b** and SiO_2_ modified with MAO (at [Zr]:[Al] = 1:2600, the activity was 5.16 · 10^6^ g_PE_ mol^−1^ h^−1^). The activity of zirconocene **40** in the polymerization reaction on a MAO-SiO_2_ carrier was 2.15·10^6^ g_PE_ mol^−1^ h^−1^ (M_W_= 229,500). The reactivity of complex **132c** ((Me_3_SiCp)_2_ZrH_3_AlH_2_) activated by MAO and supported on SiO_2_ with the addition of molecular H_2_ increased by 25% during ethylene polymerization. Nevertheless, there was a significant decrease in the molecular weight of the product from M_N_ = 63,700 and M_W_ = 175,000 to M_N_ = 691 and M_W_ = 1930 with the introduction of H_2_, which was used as a chain transfer agent in the reactions of ethylene polymerization and copolymerization of ethylene with 1-hexene [121].

Polyethylene was synthesized in the presence of the Bu^n^Cp_2_ZrH_3_AlH_2_/MAO/KCl system that showed activity at a level of 4.07 · 10^6^ g_PE_ mol^−1^h^−1^ (M_W_ = 16,950) [123].

**Scheme 66:**
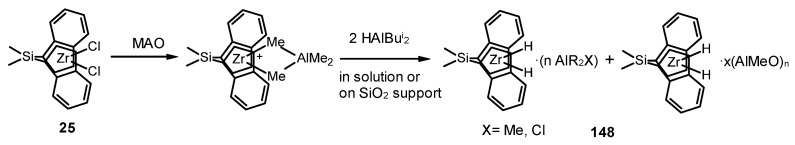
Reaction of (SBI)ZrCl_2_ with MAO in the presence of HAlBu^i^_2_ or AlBu^i^_3_ [84].

The neutral dihydride complexes, (SBI)ZrH_2_·{nAlR_2_X} (**148**), were found as a result of the interaction between (SBI)ZrCl_2_ (**25**) and MAO ([Al]MAO/[Zr] = 600) both in the solution and on the surface of SiO_2_ in the presence of diisobutylaluminum hydride or triisobutylaluminum (Scheme 66) [84]. The ^1^H NMR spectrum of the systems based on (SBI)ZrCl_2_, Al_2_Me_6_, HAlBu^i^_2_, and MAO contained the broadened singlet signals of a Zr–H bond at δ_H_ −1.39 (SBI)ZrH_2_·{2AlMe_3_}), −1.54 (SBI)ZrH_2_·{2AlMe_2_Cl}), and −1.95 ppm (SBI)ZrH_2_·{x(AlMeO)_n_}) (**148**). The observed complexes appeared to be inactive in olefin polymerization.

**Scheme 67:**
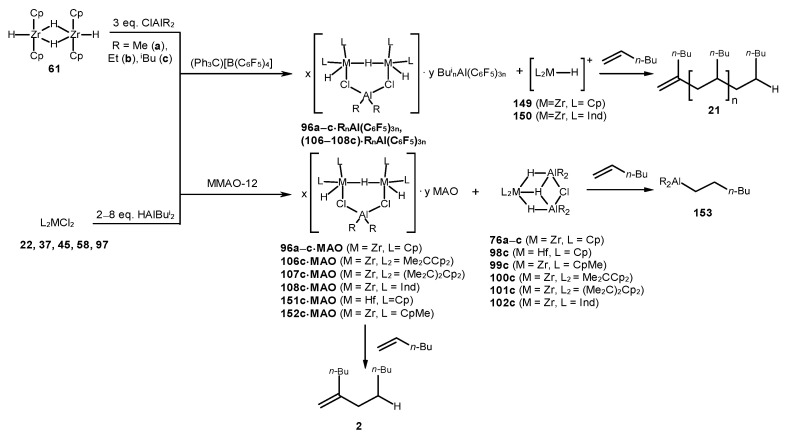
Structures observed in the Cp_2_ZrH_2_-ClAlR_2_-MMAO-12 ((Ph_3_C)[B(C_6_F_5_)_4_]) and L_2_ZrCl_2_-HAlBu^i^_2_-MMAO-12 ((Ph_3_C)[B(C_6_F_5_)_4_]) systems and reactivity of Zr,Al–hydride intermediates towards an alkene [71,72,73,74,75].

The addition of MMAO-12 to the Cp_2_ZrH_2_-ClAlR_2_ system (R = Me (**a**), Et (**b**), and Bu^i^ (**c**)) in a [Zr]:[ClAlR_2_]:[MMAO-12] ratio of 1:(1.5–3):(3–8), containing equilibrium mixture of complexes **75a**–**c**, **76a**–**c**, and **96a**–**c** (Scheme 52), led to the appearance of adduct **96a**–**c** with MMAO-12 with the separation of the reaction mixture into light and heavy fractions (Scheme 67) [71,72,73,74,75]. The triplet signals of protons of a Zr-H-Zr bond at δ_H_ −6.56–−6.44 ppm and doublet signals of a Zr-H-Al fragment at δ_H_ from −1.74 to −1.28 ppm were observed in the ^1^H NMR spectrum of the light fraction in the case of the **96a**–**c·MAO** adduct. The ^1^H NMR spectrum of the heavy adduct, **96a**–**c·MAO**, exhibited the broadened signals of hydride atoms in the ranges of δ_H_ −7.10–−6.54 ppm (Zr-H-Zr) and δ_H_ −1.44–−1.22 ppm (Zr-H-Al). When (Ph_3_C)[B(C_6_F_5_)_4_] was added to the [Cp_2_ZrH_2_]_2_-ClAlEt_2_ system (at a ratio of 1:(3–4):(0.15–0.5)), additional upfield triplet and doublet signals at δ_H_ −6.87 ppm and −1.72 ppm, respectively, appeared in the ^1^H NMR spectrum, which were assigned to the **96b·R_n_Al(C_6_F_5_)_3−n_** adduct [72]. Similar adducts were found in the Cp_2_ZrCl_2_-HAlR_2_-MMAO-12 catalytic systems ((Ph_3_C)[B(C_6_F_5_)_4_]) [71,72,73].

Analogous MMAO-12 associations ((**106**–**108c**)**·MAO**, **151c·MAO**, **152c·MAO**) were observed in the reactions of L_2_ZrCl_2_ (**22**, **37**, **45**, **58**, **97**) with HAlBu^i^_2_ and MMAO-12 (Scheme 67) [74,75]. Moreover, complexes, probably being a cationic type, [Cp_2_ZrH]^+^ (**149**, **150**), whose proton signals were located at δ_H_ −6.6–−0.1 ppm in the ^1^H NMR spectra, formed in the L_2_ZrCl_2_-HAlBu^i^_2_-(Ph_3_C)[B(C_6_F_5_)_4_] catalytic systems (L = Cp, Ind) at a [Zr]:[Al]:[B] ratio of 1:(5–8):0.5 (Scheme 67) [71,72,74].

An NMR study on the activity of the Zr,Al–hydride intermediates towards an alkene (Scheme 67) showed that hydride complexes **76a**–**c** and **98c**–**102c** reacted first to provide hydrometalation product **153**. Intermediates with the [(L_2_Zr)_2_H_3_] moiety associated with MAO or R_n_Al(C_6_F_5_)_3−n_ provided dimer **2**. The addition of an alkene to the systems with hydride species of a cationic type, [L_2_ZrH]^+^ (**149c** or **150c**), led to the formation of oligomer **21** at a high rate **[71,72,74,75]**. As a result, studies on the metallocene–OAC–activator systems (MMAO-12, (Ph_3_C)[B(C_6_F_5_)_4_]), disclosed the generation of various hydride clusters, including bis-zirconium hydride intermediates of the [(L_2_Zr)_2_H_3_] type, which were the precursors of active centers that initiated an alkene dimerization, whereas cationic species [L_2_ZrH]^+^ ensured the formation of the oligomeric products.

## 4. Conclusions

Dimerization and oligomerization reactions are widely used to convert light olefins resulting from various processes (thermal and catalytic cracking, Fischer–Tropsch synthesis, etc.) into higher olefins that are demanded in various industrial fields. The dimerization and oligomerization of α-olefins are carried out by heterogeneous acid catalysis, which is mainly used for the production of fuels, and by transition metal catalytic systems, utilized primarily for the production of high value-added products. 

An analysis of the data in the literature shows that much attention is commonly paid to the consideration of the catalytic properties of transition metal complexes of various structures and systems to search for the most active catalysts for alkene dimerization and oligomerization. Here, the focus is shifting towards post-metallocene complexes and heterocenes, where varying the transition metal atom and ligand structure allows a significant influence to be exerted on the activity of catalytic systems, the regioselectivity of alkene insertion, and the molecular weight distribution of reaction products. The design of new activators with predictable effects in contrast to stochastic-structured aluminoxanes is highly relevant, as well as the search of heterogeneous carriers for catalytic systems, aiming to implement new processes on an industrial scale. Another interesting direction is the variation of monomer structures (including the use of polar monomers) to obtain products with unique physical and physicochemical properties for the development of new materials.

Metal hydrides can act as active centers of the considered catalytic systems. The metal-H bond exhibits remarkable activity, contributing to a diverse array of catalytic applications. This includes the reduction in unsaturated compounds; the di-, oligo-, and polymerization of alkenes with varied structures; as well as the functionalization of olefins and acetylenes through hydrometalation. Despite the large amount of information on the structures of hydride intermediates generated in transition metal complex activator systems, the mechanisms of their actions in the discussed processes remain open questions.

Therefore, the investigation of the mechanisms of alkene dimerization and oligomerization reactions is crucial for a more targeted exploration of novel, efficient catalysts and activators. In this field, significant attention will still be given to metallocene systems as the most convenient models for studying reaction mechanisms. Priority lies in comprehending the structure and dynamics of active centers, a factor that is significantly influenced by the metal’s nature, ligand, and cocatalyst structure. The σ- and π-ligand environments of the transition metal play pivotal roles in determining the lifespan of specific active sites, which are necessary for successful alkene insertion, chain propagation, and termination. Consequently, future research demands a comprehensive approach encompassing the exploration of catalytic system properties and the experimental and theoretical analysis of structural and dynamic features of hydride intermediates. This holistic approach aims to develop robust models for reaction mechanisms and predict the properties of new promising catalytic systems.

## Data Availability

Not applicable.
